# Parvalbumin and GABA Microcircuits in the Mouse Superior Colliculus

**DOI:** 10.3389/fncir.2018.00035

**Published:** 2018-05-04

**Authors:** Claudio A. Villalobos, Qiong Wu, Psyche H. Lee, Paul J. May, Michele A. Basso

**Affiliations:** ^1^Fuster Laboratory of Cognitive Neuroscience, Department of Psychiatry and Biobehavioral Sciences – Department of Neurobiology, Semel Institute for Neuroscience and Human Behavior – Brain Research Institute, David Geffen School of Medicine, University of California, Los Angeles, Los Angeles, CA, United States; ^2^Department of Neurobiology and Anatomical Sciences, University of Mississippi Medical Center, Jackson, MS, United States

**Keywords:** superior colliculus (SC), parvalbumin, mouse, optogenetics, GABAergic neurons, orienting movements, attention

## Abstract

The mammalian superior colliculus (SC) is a sensorimotor midbrain structure responsible for orienting behaviors. Although many SC features are known, details of its intrinsic microcircuits are lacking. We used transgenic mice expressing reporter genes in parvalbumin-positive (PV^+^) and gamma aminobutyric acid-positive (GABA^+^) neurons to test the hypothesis that PV^+^ neurons co-localize GABA and form inhibitory circuits within the SC. We found more PV^+^ neurons in the superficial compared to the intermediate SC, although a larger percentage of PV^+^ neurons co-expressed GABA in the latter. Unlike PV^+^ neurons, PV^+^/GABA^+^ neurons showed predominantly rapidly inactivating spiking patterns. Optogenetic activation of PV^+^ neurons revealed direct and feedforward GABAergic inhibitory synaptic responses, as well as excitatory glutamatergic synapses. We propose that PV^+^ neurons in the SC may be specialized for a variety of circuit functions within the SC rather than forming a homogeneous, GABAergic neuronal subtype as they appear to in other regions of the brain.

## Introduction

The mammalian superior colliculus (SC) is a midbrain structure specializing in translating sensory information into commands for orienting movements and redirecting attention (reviewed in, Basso and May, 2017). With the development of new molecular tools and transgenic mouse lines, we are now well-poised to unravel the neuronal microcircuits within the SC underlying these complex behaviors. Here, we took advantage of transgenic mice expressing a reporter gene in neurons containing the Ca^2+^-binding protein, parvalbumin (PV) and mice expressing the light-activated ion channel channelrhodopsin-2 (ChR2) in molecularly identified parvalbumin-positive (PV^+^) neurons to determine the features and influence of PV^+^ neuronal activation on SC microcircuits.

In all areas of the cerebral cortex studied thus far, as well as in hippocampus and striatum, PV appears almost exclusively in a subpopulation of GABAergic neurons (Gonchar et al., 2008; Klausberger and Somogyi, 2008; Tremblay et al., 2016). In cortex, these GABAergic neurons exhibit fast-spiking patterns and form direct inhibitory synapses with the somata, proximal dendrites, and initial segments of cortical pyramidal neurons (Kawaguchi et al., 1987; Kawaguchi, 1993; Kawaguchi and Kubota, 1993, 1997; Gupta, 2000; Markram et al., 2004; Taniguchi et al., 2011; Taniguchi, 2014; Tremblay et al., 2016). These GABAergic PV^+^ neurons thus play a key role in intrinsic inhibitory microcircuits in cortical and subcortical regions (Cardin et al., 2009; Sohal et al., 2009; Chen et al., 2017). Impairments of inhibitory processes related to the function of PV^+^ neurons are associated with disorders ranging from autism to schizophrenia, highlighting the importance of PV^+^ neurons in mechanisms underlying higher cognitive functions (Lewis et al., 2012; Wöhr et al., 2015; Chung et al., 2016; Hashemi et al., 2017; Kim et al., 2017). We sought to determine whether PV^+^ neurons play a similar role in SC circuits.

The mammalian SC is a layered structure. The superficial layers (sSC) participate in visual functions and the intermediate layers (iSC) contribute to the control of orienting behaviors; thus, we often refer to these as visuosensory and motor layers, respectively. The sSC contains neurons expressing a variety of Ca^2+^-binding proteins, including PV (Behan et al., 1992; Mize et al., 1992; Leuba and Saini, 1996; Lee et al., 2006). Specific calcium binding properties may characterize individual streams for SC visual information processing. For example, calbindin^+^ neurons in sSC receive inputs from K-type ganglion cells, whereas PV^+^ neurons receive input from M-type ganglion cells, as well as from cortical neurons with M-like properties (Mize et al., 1991; Casagrande, 1994; Mize, 1999). These two types of neuron inhabit different strata within the sSC, with PV^+^ neurons lying in the lower superficial layer (SGS) and stratum opticum (SO) (Illing et al., 1990). These PV^+^ neurons in turn, project to the lateral posterior nucleus of the thalamus, indicating that at least some PV^+^ neurons in the SC are projection neurons, as opposed to interneurons (Casagrande, 1994; Mize, 1996). A recent report using transgenic mouse models provides further support for the idea that PV^+^ neurons in the sSC form extrinsic excitatory circuits (Shang et al., 2015). These authors discovered that sSC PV^+^ neurons form excitatory connections with the parabigeminal nucleus, which in turn projects to the amygdala. Activation of this pathway produces fear responses, including escape and freezing behaviors. Together, the work in sSC suggests that collicular PV^+^ neurons play a different role than that proposed for PV^+^ local circuit neurons in the cerebral cortex.

We designed a series of experiments to test the hypotheses that PV^+^ neurons in the SC can form intralaminar inhibitory circuits, in addition to their known role in extrinsic excitatory circuits. Inhibitory microcircuits modulate the flow of retinal information from the sSC to the iSC (Hall and Lee, 1997; Lee et al., 1997; Isa et al., 1998) and are thought to play a key role in circuit interactions leading to eye movement choices (reviewed in, Basso and May, 2017). Therefore, understanding how inhibitory neuronal microcircuits act within the SC will provide valuable insight into understanding how the brain controls behavior. To this end, we performed immunohistochemical experiments comparing PV and GABA expression throughout the rostral-caudal extent of the SC. In brain slices from transgenic mice expressing a reporter gene (tdTomato) in PV^+^ neurons, we characterized the morphology, as well as the biophysical and electrophysiological properties of SC PV^+^ neurons. We crossed the Ai9;PV-Cre mouse line with the GAD67-EGFP knock-in mouse to create a new mouse expressing both tdTomato and EGFP reporters exclusively in PV^+^/GABA^+^ neurons and assessed the distribution and electrophysiological properties of these neurons throughout the SC. Finally, we crossed the PV-IRES-Cre mouse line with the Ai32 mouse line to express ChR2 in PV^+^ neurons. Using optogenetic stimulation, we tested the functional connectivity of the synapses formed by PV^+^ neurons within the SC. Our results demonstrate that PV^+^/GABA^+^ neurons exist in the SC, form functional inhibitory circuits within the SC, and show physiological properties that differ from those in cerebral cortex, hippocampus and striatum. We conclude that PV^+^ neurons in the SC likely play different roles in collicular function than they do in cortex.

## Materials and Methods

All experimental protocols involving animals were approved by the UCLA Chancellor’s Animal Research Committee and complied with standards set by the Public Health Service policy on the humane care and use of laboratory animals, as well as all state and local guidelines. To identify parvalbumin containing neurons (PV^+^) in the SC, we used Ai9;PV-Cre transgenic mice generated from crossing of PV-IRES-Cre knock-in females (Jackson Laboratories, stock no. 008069) and male Ai9-tdTomato reporter knock-in mice (Jackson Laboratories, stock no. 007905). All experimental mice were heterozygous for the transgenes on a C57BL/6 background. To identify PV^+^ and GABA^+^ neurons in the SC, we used an Ai9;PV-Cre;GAD67 transgenic mice generated by crossing Ai9;PV-Cre females and GAD67-GFP males (Jackson Laboratories, stock no. 007677). These experimental mice were homozygous for tdTomato, heterozygous for PV-Cre and hemizigous for GAD67-GFP on a C57BL/6 background. Optogenetic experiments were carried out using Ai32;PV-Cre mice generated from PV-IRES-Cre knock-in female mice crossed with Ai32 males [Ai32(RCL-ChR2(H134R)/EYFP); Jackson Laboratories, stock no. 012569].

### Immunohistochemistry

Ai9;PV-Cre mice brain sections were used to determine the co-expression of PV and GABA in neurons of the SC. Mice were deeply anesthetized with isoflurane/O_2_ (1 L/min) and perfused transcardially with 4% paraformaldehyde in 0.12 M phosphate buffer (PB), pH 7.3. The brains were removed and post-fixed for 2 h in 4% paraformaldehyde PB. After rinsing, the brains were cryoprotected in a 30% sucrose phosphate buffer saline (PBS) overnight at room temperature, embedded in Tissue-Tek^®^ optical cutting temperature compound (Sakura Finetek), frozen on dry ice, and cryo-sectioned coronally (30 μm thickness). Sections were then labeled with antibodies for GABA (Sigma A2052). Briefly, sections were rinsed in 0.1M Tris-buffered saline (TBS) and then incubated in TBS containing 5% donkey serum and 1% Triton X-100 for 2 h to block non-specific staining and enhance penetration. The tissue was rinsed three times with TBS between all subsequent steps in the immunohistochemical procedures. Sections were incubated in primary antibody, rabbit anti-GABA (1:1000), overnight at room temperature. Next, the tissue was incubated overnight at room temperature in a secondary antibody, donkey anti-rabbit IgG conjugated with Alexa Fluor 488 (1:500) (Jackson ImmunoResearch), to which Neurotrace^®^ (1:500) (Thermo Fisher Scientific) was added as a counterstain. After thorough rinsing, sections were mounted on slides and cover-slipped with Prolong Diamond Antifade mounting medium (P36970). All sections were scanned with a LSM 800 (Carl Zeiss) confocal microscope, and confocal images were analyzed with Zen 2.3 lite imaging software (Carl Zeiss).

### Neuronal Quantification

Processed tissue was scanned for neurons in the superficial and intermediate layers of the SC (sSC and iSC, respectively), as well as for neurons in primary visual cortex (Cx). Z-stack images (18–25 sections, ∼0.6 μm thickness) were acquired from the sSC and iSC at locations across the mediolateral extent of the layers containing abundant PV^+^ neurons. Images were taken while alternating between three channels (excitation spectra 488, 561, and 640 nm for PV^+^, GABA^+^, and Neurotrace labels, respectively) throughout the Z-stack. Images from three to six regions from either rostral, middle or caudal SC were obtained from four mice (255 μm × 255 μm) and single- and double-labeled neurons, positive for either tdTomato and/or the immunohistochemical label for GABA, were identified and quantified within the selected regions. Verification of immuno-positive neurons was done by sliding through the optical z-sections, and all PV^+^ cells were determined to be neurons by counterstaining with Neurotrace^®^ (**Supplementary Figure S1**). Specifically, under the tdTomato filter and corresponding excitation spectra, PV^+^ neurons were individually identified in each of the z-stack images of a section. Then, under the AF488 filter and excitation, each of the previously selected neurons was checked for GABA labeling. In this manner, PV^+^ or PV^+^/GABA^+^ neurons were quantified in each of the sections. Quantification of double-labeled neurons from sections through cortex processed at the same time were used as a control.

### Acute Brain Slice Preparation

Coronal brain slices of 250–300 μm in thickness that included the SC were prepared as follows: mice (P30-45) were anesthetized with isoflurane, decapitated and their brains were quickly removed and cooled (4°C) in high sucrose artificial cerebrospinal fluid (sucrose ACSF) containing (in mM): 240 sucrose, 7 D-glucose, 7 MgCl_2_, 1.25 NaH_2_PO_4_, 2.5 KCl, 25 NaHCO_3_, 0.5 CaCl_2_, that was bubbled to saturation with 95% O_2_ – 5% CO_2_. The isolated brain was then affixed to the specimen plate using cyanoacrylate glue, and cut using a vibratome (Leica VT 1200S). Coronal slices were subsequently transferred to a recovery chamber containing standard ACSF (in mM: 126 NaCl, 2.5 KCl, 26 NaHCO3, 1.25 NaH2PO4, 2 CaCl_2_, 2 MgCl_2_, and 10 glucose) at 35°C for at least an hour before recording.

### Electrophysiological Recordings

x Brain slices were transferred to a submerged recording chamber on the stage of an upright Zeiss (Axio Examiner D1) or a Nikon (Eclipse E600) microscope. Slices were superfused (2–3 ml/min) with standard ACSF and maintained at ∼30°C using an in-line heater controller (TC-324C, Warner Instruments). Individual PV^+^ or PV^+^/GABA^+^ neurons from the sSC or iSC were identified under DIC or fluorescence with a Hamamatsu Cooled CCD camera (C11440-42U). Recordings were obtained using 3–4 MΩ electrodes filled with intracellular solution (in mM: 150 K-Gluconate, 15 KCl, 1.5 MgCl_2_, 10 HEPES, 0.1 EGTA, 2 Na-ATP, and 0.5 Na-GTP; 290 mOsm; pH 7.3). In some cases, recorded neurons were labeled by injecting either Lucifer Yellow or biocytin added to the intracellular solution. Signals were amplified using a Multiclamp 700B amplifier (Molecular Devices, San Jose, CA, United States), digitized and stored on a PC. Series resistance and whole-cell capacitance were automatically compensated. Total resistance values (*R*_t_ = *R*_a_ + *R*_m_) where *R*_t_ is total resistance, *R*_a_ is access resistance and *R*_m_ is membrane resistance, for each recording was obtained immediately after breaking into the neuron from the Membrane Test Window of the *Clampfit 10* software. The *R*_t_ values are calculated based on a series of known voltage pulses applied to the neuron and measuring the corresponding current values obtained right after breaking the gigaseal. All electrophysiological data were analyzed using the *Clampfit 10* software. All chemicals were purchased from Sigma-Aldrich. Optogenetic experiments were carried out using brain slices obtained from the Ai32;PV-Cre mice. We visualized PV^-^ neurons with infrared differential interference contrast (IR-DIC) microscopy. We excited PV^+^ neurons by illuminating the SC through the objective with a 470 nm LED light (Mightex). To prevent firing of action potentials, QX-314 (2.5 mM) was added to the intracellular solution. Traces recorded from collicular PV^-^ neurons depicting postsynaptic currents are shown as the averaged responses of three consecutive light pulses. All recorded neurons were deemed healthy by assessing *V*_m_ magnitude and stability, and maximum spike voltage (e.g., < -50 mV and >0 mV, respectively).

### Morphology

To identify and categorize the morphology of PV^+^ or PV^+^/GABA^+^ neurons in the SC, individual neurons were patched and recorded using pipettes filled with either Lucifer Yellow CH dipotassium (1 mg/ml, Sigma) or biocytin 0.5% added to the intracellular solution. Recordings were made from acute slices obtained from either Ai9;PV-Cre or Ai9;PV-Cre;GAD67 mice to assess the morphology of PV^+^ and PV^+^/GABA^+^ neurons, respectively. After 10–30 min, the slices were fixed with 4% paraformaldehyde for 24 h and then stored in a 0.1M PBS/0.06% NaN_3_ solution. After three rinses with PBS, the slices were incubated with streptavidin conjugated with Alexa Fluor 647 (1:500) (Thermo Fisher Scientific S32357) overnight at room temperature. After thorough rinsing, slices were mounted on slides and cover-slipped with Fluoro-Gel in TES buffer mounting medium (Electron Microscopy Sciences). Images of the recorded neurons were obtained using a LSM 800 (Carl Zeiss) confocal microscope.

Classification of neuronal morphology is a daunting task due to the fact that different authorities have used different terminologies and focused on different characteristics. In addition, different methodologies including Golgi staining (Langer and Lund, 1974; Norita, 1980; Ma et al., 1990), *in vivo* intracellular staining and serial reconstruction (Mooney et al., 1985; Moschovakis and Karabelas, 1985; Moschovakis et al., 1988a,b), and *in vitro* intracellular staining (Hall and Lee, 1997; Edwards et al., 2002; Lee et al., 2007) provide different types of information, and these studies have been performed in a variety of species, e.g., mouse, hamster, cat, and monkey. For this study, we adopted a simplified classification system similar to that of Edwards et al. (2002). In the sSC, we used the term narrow field vertical for any neurons whose dendritic fields were taller than were wide and <100 μm across. This included classical narrow field vertical neurons, which displayed long apical dendrites and short basal dendrites, as well as neurons which had primary dendrites extending at a variety of points from their somata, but which nevertheless had a columnar dendritic field shape. This category also included up-side-down examples where the soma was near the surface and most of the dendritic field extended ventrally. We used the term horizontal, for neurons in which the dendritic field was clearly non-symmetrical, with the long axis oriented parallel to the collicular surface. These neurons had either fusiform or spherical somata, and they sometimes had primary dendrites that initially extended in the dorsoventral direction, but which then turned parallel to the surface. We used the term wide field vertical, for neurons whose dendritic fields, unlike those of the horizontal neurons, extended toward the collicular surface. This type of neuron was discriminated from narrow field vertical type by the fact that the dendritic field was greater than 150 μm across. (It should be noted that due to the fact these were stained in slices, we could not see the entire extent of their dendritic field.) In theory, this class includes pyriform neurons (Langer and Lund, 1974), having funnel-shaped dendritic fields and no basal dendrites, that have been described previously (Mooney et al., 1985; Luksch et al., 1998; Lee et al., 2001; May, 2006). In the present case, only non-pyriform neurons that displayed both dorsally and ventrally extending dendrites were observed. The final sSC category that we observed were stellate neurons. Stellate neurons were characterized by primary dendrites that extended in all directions from the soma and which formed a roughly symmetric dendritic field with no obvious orientation. Using this neuronal classification scheme, we observed four different neuronal types within the sSC, as did a previous study using a cell-type clustering analysis of filled neurons (Gale and Murphy, 2014). The iSC contains a number of different multipolar cell types (Norita, 1980; Ma et al., 1990). In the present experiment, we divided the stained neuronal population into just two classes: horizontal and radial-stellate. The iSC horizontal neuronal population had essentially the same features as those seen in the sSC. The iSC radial-stellate neurons had roughly spherical dendritic fields like sSC stellates. However, iSC radial-stellate dendritic fields were much larger and the dendrites branched far less than sSC stellate neurons. Neurons of this type have been variously termed small multipolar, stellate and radial by others (Langer and Lund, 1974; Norita, 1980; Laemle, 1981).

## Results

### Parvalbumin-Positive (PV^+^) Neurons Co-localize GABA in the SC

**FIGURE 1:**
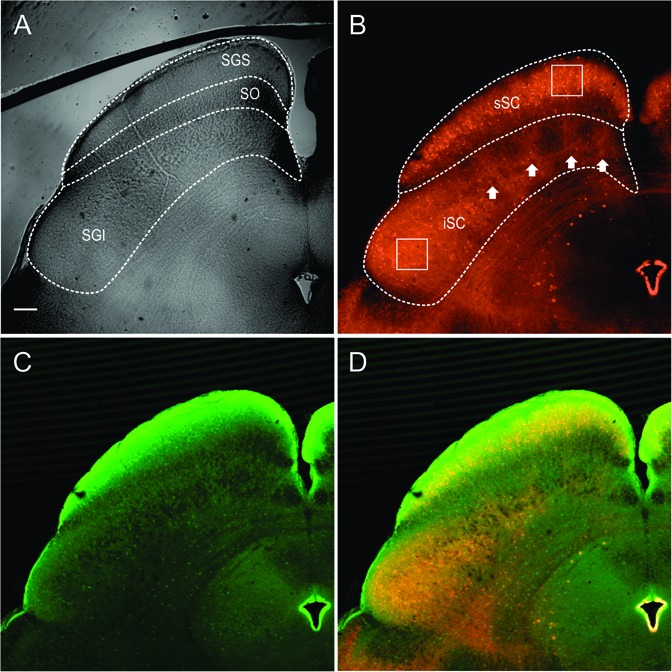
Distribution of PV and GABA in the Ai9;PV-Cre mouse SC. **(A)** Differential interference contrast (DIC) image from an Ai9;PV-Cre mouse showing the left side of a 300 μm slice through the SC and the layers (SGS; stratum griseum superficiale; SO, stratum opticum; sSC, superficial/visuosensory layers; iSC, intermediate/motor layer; SGI, stratum griseum intermediale). **(B)** Confocal image of the same section showing the distribution of PV^+^ neurons and neuropil in both layers of the SC as visualized by tdTomato fluorescence. Squares on sections within the layers are shown in expanded view in **Figure 2A**. White arrows highlight the patchy distribution of PV^+^ labeling in iSC. **(C)** Confocal image depicting the distribution of GABA^+^ neurons and neuropil in the same section detected by GFP immunofluorescence using anti-GABA antibody. **(D)** Merged PV^+^ and GABA^+^ confocal images **(B,C)**. Scale bar: 200 μm.

The stratum zonale, the stratum griseum superficiale (SGS) and the SO comprise the superficial, visuosensory layers of the SC (sSC), and below these layers is the stratum griseum intermediale, which we refer to as the intermediate or motor layers (iSC). **Figure 1A** shows the SC viewed with differential interference contrast (DIC) microscopy, as it appears in the recording chamber. The borders of the layers are demarcated with dashed white lines. **Figure 1B** shows the distribution of PV-positive (PV^+^) neurons in the SC from the Ai9;PV-Cre mouse. Most of the red (tdTomato) PV^+^ neurons and neuropil in the sSC lie in a band located in the lower portion of the SGS. Scattered neurons are present dorsal to this lamina and ventrally, in SO. PV expression also appears throughout the iSC, and it includes both neurons and neuropil (**Figure 1B**). Unlike the sSC laminar pattern, the iSC pattern is patchy, with the densest labeling in the lateral-most aspect. The appearance of the PV labeling in the Ai9;PV-Cre mouse (**Figure 1B**, white arrows), is similar to that observed in rats using immunohistochemistry (Illing et al., 1990).

To investigate whether PV^+^ neurons in the SC also express gamma-aminobutyric acid (GABA) as they do in cerebral cortex (Celio, 1986), we used an antibody to GABA on sections from four Ai9;PV-Cre mice. **Figure 1C** shows the distribution of GABA labeling from the same section shown in **Figure 1B**. GABA antibody densely labels the SC, primarily in the upper superficial layer with comparatively less label observed in SO and in iSC. The lower SGS lamina seen with PV staining is not evident. A merged view of the PV^+^ and GABA^+^ images (**Figure 1D**) shows that double-labeled neurons are sparsely distributed in the two layers.

**FIGURE 2:**
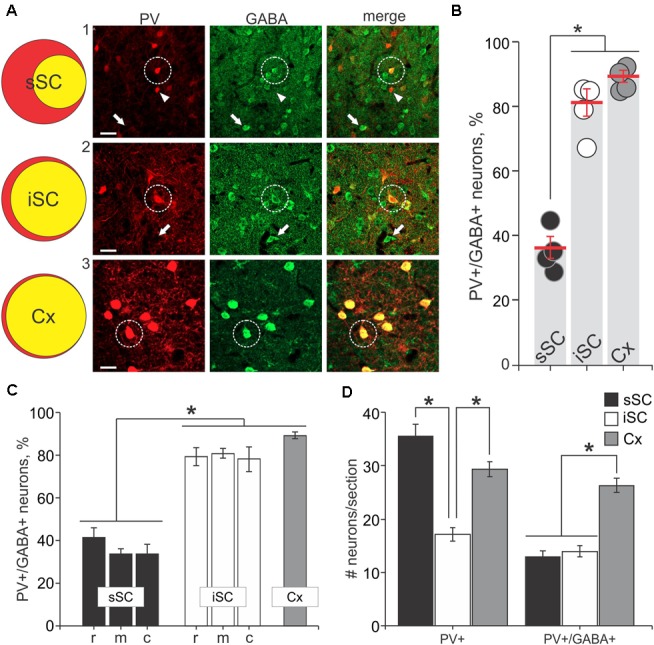
PV^+^ neurons contain GABA^+^ in the SC. **(A)** Far left, Venn diagrams representing the proportion of PV^+^/GABA^+^ neurons (yellow) of the total number of PV^+^ neurons (red) from sections of sSC, iSC and V1 of cerebral cortex (Cx). Right panels, confocal images of sections from sSC, iSC, and Cx (row **A_1-3_**, respectively) showing PV^+^ (left column), GABA^+^ (middle), and the merged images (right). White arrowheads indicate examples of PV^+^ neurons. White arrows show examples of GABA^+^ neurons and white circles highlight PV^+^/GABA^+^ neurons. Scale bar: 20 μm. **(B)** Percentage of PV^+^/GABA^+^ neurons of the total number PV^+^ neurons counted in sSC, iSC, and Cx for each of the mice (*n* = 4). Red line, mean ± SE. **(C)** Mean percentage of double-labeled PV^+^/GABA^+^ neurons in sections from sSC (black), iSC (white) obtained from rostral (r), middle (m) or caudal (c) SC brain slices and from Cx (gray). ^∗^*p* < 0.05, Kruskal–Wallis. Bar: mean ± SE. **(D)** Mean ± SE number of PV^+^ and double labeled PV^+^/GABA^+^ neurons per section (4–8 sections/mouse) from sSC, iSC, and Cx. ^∗^*p* < 0.05, Mann–Whitney.

The appearance of double label in the section shown in **Figure 1D** was consistent across our sample of four mice. We obtained 40× magnification confocal images of similar sections throughout the sSC and the iSC, and performed counts of the yellow neurons containing both tdTomato and GABA. **Figure 2A** shows example images taken from regions of the sSC (A_1_) and iSC (A_2_) marked by the white squares in **Figure 1B**, and a similarly sized region of visual cortex (A_3_; Cx). In both layers, we observed singly labeled GABA^+^ and PV^+^ neurons (**Figure 2A**, arrows and arrowheads, respectively) and double labeled PV^+^/GABA^+^ neurons (**Figure 2A**, circles). In visual cortex, the majority of PV^+^ neurons expressed GABA (**Figure 2A_3_**). The Venn diagrams in **Figure 2A** show schematically what is quantified in **Figure 2B** based on sample counts from 13 to 20 brain sections from four mice. The number of neurons co-localizing PV and GABA with respect to the total PV^+^ neuronal population (36 ± 3% sSC, 81 ± 4% iSC, and 90 ± 2% Cx; ^∗^*p* < 0.05, paired *t*-test), demonstrates clear differences in the proportions of PV^+^ neurons expressing GABA (**Figure 2B**). These differences remain when sorting the slices into rostral, middle and caudal collicular regions (**Figure 2C**). Like the cortex, iSC had a high proportion of PV^+^/GABA^+^ neurons, although the overall number of PV^+^ neurons in the iSC was significantly less than found in sSC and Cx (**Figure 2D**; ^∗^*p* < 0.05, Mann–Whitney).

### Physiological and Morphological Features of PV^+^ Neurons in the SC

**FIGURE 3:**
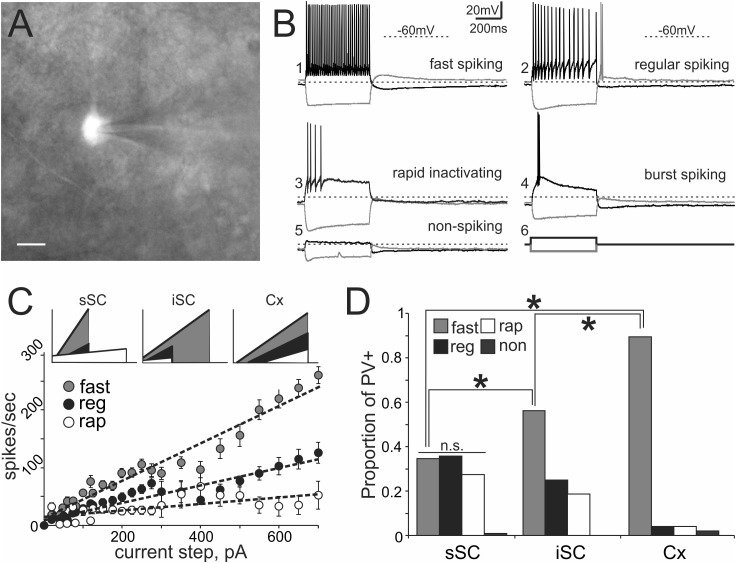
PV^+^ neurons in the SC show a variety of electrophysiological properties. **(A)** Visually identified and recorded PV^+^ neuron from a brain slice of an Ai9;PV-Cre mouse. Scale bar: 10 μm. **(B)** Representative traces from whole-cell patch-clamp recordings obtained from PV^+^ neurons in the SC. **(B_1_)** Shows fast spiking, **(B_2_)** regular spiking, **(B_3_)** rapidly inactivating, **(B_4_)** burst spiking and **(B_5_)** non-spiking. Gray and black traces represent voltages resulting from injection of negative and positive step currents (–100 pA/+100 pA, **B_6_**), respectively. Dotted line, –60 mV applies to all five traces. Scale bar: 20 mV/200 ms. **(C)** Mean values of step current vs. spikes/sec (I/F) curves calculated from all recorded PV^+^ neurons showing the most common spiking classes; fast (gray circles), regular (black circles) and rapidly inactivating (open circles). Dotted lines represent the linear regression computed for each of the classes. The I/F plots at the top provide a comparison of the linear regressions calculated for fast (gray), regular (black), and rapidly inactivating neurons (white) recorded from sSC (left), iSC (middle), and Cx (right). **(D)** Proportion of fast, regular, rapidly inactivating and non-spiking PV^+^ neurons recorded in sSC, iSC, and Cx. ^∗^*p* < 0.05, *z*-test.

Transgenic mouse lines allow for targeted recordings of specific visually identified neurons. In cerebral cortex, PV^+^ neurons are known to be GABA^+^ and fast-spiking, with high-frequency trains of action potentials that display little adaptation (Kawaguchi et al., 1987; Cauli et al., 1997; Kawaguchi and Kubota, 1997; Xu and Callaway, 2009; Taniguchi, 2014). We asked whether PV^+^ neurons in the colliculus show similar characteristics by recording visually identified PV^+^ neurons in whole-cell configuration from slices obtained from Ai9;PV-Cre mice (**Figure 3A**). **Figure 3B** shows representative traces obtained from PV^+^ neurons in response to +100 pA (black) and -100 pA (gray) current injections. **Table 1** reports electrophysiological measurements from our sample of neurons. We found that PV^+^ neurons in the SC showed a variety of firing profiles, including fast-spiking (**Figure 3B_1_**), regular spiking (**Figure 3B_2_**) rapidly inactivating (**Figure 3B_3_**), burst spiking (**Figure 3B_4_**), and non-spiking (**Figure 3B_5_**) patterns. Thus, in contrast to cerebral cortex, where most PV^+^ neurons are fast-spiking, SC PV^+^ neurons exhibit heterogeneous electrophysiological properties.

**Table 1:** Electrophysiological parameters from PV^+^ neurons in the SC.

		V_m_	R_t_	C_m_	sAHP	I_h_
**sSC**	Reg	60.64 ± 1.12	293.79 ± 41.24	53.62 ± 8.17	-1.30 ± 1.54	4.46 ± 0.74*
	Rap	60.76 ± 2.13	415.56 ± 56.8	37.81 ± 3.83	0.22 ± 0.66	3.43 ± 1.04*
	Fast	58.39 ± 1.36	338.17 ± 59.09	37.84 ± 5.9	1.86 ± 1.53	7.08 ± 1.33*
**iSC**	Reg	61.27 ± 2.11	412.88 ± 39.21	44.34 ± 6.69	2.50 ± 1.28	5.22 ± 1.88*
	Rap	55.98 ± 3.17	432.94 ± 136.52	54.86 ± 9	-1.57 ± 0.79	0.97 ± 0.93*
	Fast	57.76 ± 1.84	410.33 ± 56.1	45.46 ± 6.43	2.76 ± 0.91	3.68 ± 0.87*
**Cx**	Reg	73.98 ± 0.22	109.25 ± 1.45*	57.12 ± 4.85	1.50 ± 0	0.9 ± 0.7
	Rap	65.1 ± 7.95	121.85 ± 41.95*	84.82 ± 0	0.30 ± 0.10	1.25 ± 1.05
	Fast	69.21 ± 0.71	135.01 ± 9.26*	60.42 ± 4.04	1.21 ± 0.58	0.74 ± 0.17

*Mean values for resting potential (V_m_), total resistance (R_t_), membrane capacitance (C_m_), slow afterhyperpolarization (sAHP), and hyperpolarization-activated current (I_h_) found in PV+ neurons in SC and Cx. ^∗^Represent whether I_h_-dependent depolarization after the current step triggered spikes in the recordings.*

To obtain an unbiased classification of SC PV^+^ neurons, we analyzed the firing pattern of each recorded neuron in response to a series of injected current amplitudes to create a series of current versus spike frequency (I/F) plots. The slope of these functions and the *R*^2^ values allowed us to assign most neurons into one of three major classes: fast-spiking, regular spiking or rapidly inactivating. **Figure 3C** depicts the mean and SE of the spike rate measured for each current step for each of the three classes of neuron. We defined fast-spiking neurons as having *a* > 150 spikes/sec rate in response to positive current steps with little spike frequency adaptation. Fast-spiking neurons (gray circles, *n* = 74) showed the steepest rate of I/F (slope = 0.33; *R*^2^= 0.94). We defined regular spiking neurons as those displaying continuous spiking in response to positive current, with marked spike frequency adaptation. Regular spiking neurons (black circles, *n* = 28) showed increasing spike attenuation, reflected as a lower slope (slope = 0.15; *R*^2^= 0.90). Rapidly inactivating neurons fired only a few action potentials in response to positive current injection. These neurons (open circles, *n* = 27) showed the lowest slope and reached a spiking plateau at currents >125 pA (slope = 0.056; *R*^2^= 0.47). The differences between the slopes were statistically significant (^∗^*p* < 0.05, Mann–Whitney) and support the classification of most PV^+^ neurons into these three classes (**Table 2**). This classification was also consistent for PV^+^ neurons across different regions: sSC, iSC, and Cx (upper plots, **Figure 3C** and **Table 3**). Finally, the three main neuron types were equally represented within the sSC (**Figure 3D**; not statistically different (n.s.); *p* = 0.23; *p* = 0.88; *p* = 0.29, *z*-test). However, the fast firing type was the most common in iSC, as seen in Cx (^∗^*p* < 0.05, *z*-test). This distribution of the various neuronal types occurred throughout the medio-lateral extent of the SC (**Supplementary Figure S2**).

**Table 2:** I/F parameters.

	Linear regression			Mean values	
	Slope	Intercept	*R*^2^	Slope	*R*^2^
Fast	0.33	11.8	0.94	0.69^∗^	0.94
Reg	0.15	11.13	0.9	0.34^∗^	0.96
Rap	0.056	14.91	0.47	0.08^∗^	0.58^∗^

*Left columns, slope, intercept and R^2^ derived from the linear regression computed from the mean I/F data for each neuronal type according to their firing patterns. Right columns, mean I/F parameters obtained from the individual slope and R^2^ values from I/F curves for each neuron. ^∗^p < 0.05.*

**Table 3:** I/F parameters.

		Linear regression			Mean values	
		Slope	Intercept	*R*^2^	Slope	*R*^2^
sSC	Fast	0.59	0.15	0.99	0.65*	0.93
	Reg	0.19	3.92	0.90	0.32*	0.92
	Rap	0.036	24.5	0.26	0.13*	0.67*
iSC	Fast	0.36	21.7	0.97	0.65*	0.97
	Reg	0.27	0.72	0.98	0.36*	0.97
	Rap	0.049	7.15	0.20	0.08*	0.62*
Cx	Fast	0.34	-11.14	0.91	0.71*	0.93
	Reg	0.25	-32.75	0.90	0.44*	0.95
	Rap	0.15	-46.41	0.70	0.11*	0.74*

*Left columns, slope, intercept and R^2^ derived from the linear regression computed from the mean I/F data for each neuronal type according to their firing patterns and location. Right columns mean I/F parameters obtained from the individual slope and R^2^ values from I/F curves for each recorded neuron. ^∗^p < 0.05.*

**FIGURE 4:**
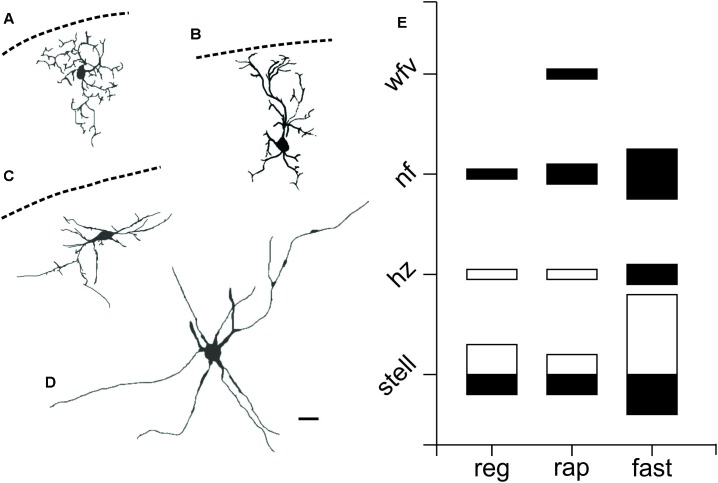
PV^+^ neurons in the SC show a variety of morphological properties. Drawings from visually identified PV^+^ neurons filled with Lucifer yellow or biocytin in the SC from slices obtained from Ai9;PV-Cre mice. **(A)** sSC stellate. **(B)** sSC narrow field vertical. **(C)** sSC horizontal. **(D)** iSC radial stellate. Scale bar: 20 μm. **(E)** Quantification of PV^+^ neurons according to their morphology and electrophysiological features for both sSC (black) and iSC (white). The size of the squares indicates number of PV^+^ neurons on each of the categories.

To investigate the morphology of PV^+^ neurons in the colliculus, we filled recorded SC PV^+^ neurons with either biocytin or lucifer yellow (*n* = 38) and classified them according to their dendritic morphology (see Materials and Methods). **Figures 4A–D** shows examples of neuronal cell types observed: stellate (**Figure 4A**), narrow field vertical (**Figure 4B**) and horizontal (**Figure 4C**) in the sSC, and radial-stellate neurons (**Figure 4D**) in iSC. Panel E summarizes the results of the morphological analysis of PV^+^ neurons in the SC with respect to their electrophysiological profiles. In the sSC, PV^+^ neurons showed a variety of morphologies, stellate, narrow field vertical, and horizontal (**Figures 4A–C,E**; black bars). Wide field vertical neurons were rarely encountered. In the iSC, the predominant morphology of PV^+^ neurons was radial-stellate, although a few horizontal neurons were encountered (**Figures 4D,E**, white bars). These results indicate that PV^+^ neurons in the sSC show a variety of morphologies and electrophysiological profiles, whereas PV^+^ neurons in the iSC are less heterogeneous. Most were fast-spiking, radial-stellate neurons (cf., **Figure 4E** black and white bars).

### Ai9;PV-Cre;GAD67 Mouse

To determine the relationship between GABA content and the physiological features of PV^+^ neurons, we crossed the Ai9;PV-Cre mouse with the GAD67-EGFP knock in mouse to create a new mouse containing both tdTomato and EGFP in PV^+^/GABA^+^ neurons. The GAD67-GFP knock in mouse labels the PV^+^ subset of GABA neurons in cerebral cortex (Chattopadhyaya, 2004). Consistent with this, the cerebral cortex of this mouse line contained only yellow neurons, indicating PV^+^/GABA^+^ co-labeling (**Supplementary Figure S3B_3_**).

**FIGURE 5:**
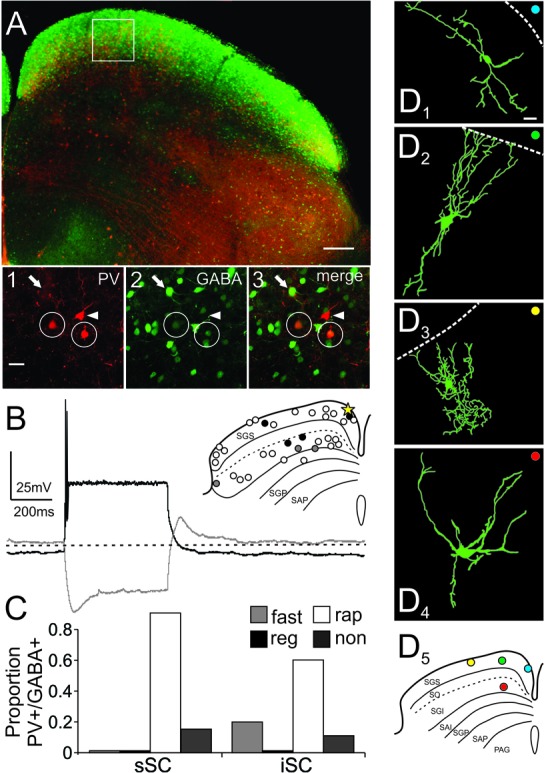
PV^+^/GABA^+^ neurons in the SC are predominantly stellate and rapidly inactivating neurons. **(A)** Low magnification confocal image of a slice from an Ai9;PV-Cre;GAD67 mouse depicting PV^+^ (tdTomato) and GABA^+^ neurons (eGFP) in the SC. Scale bar: 200 μm. White box shows the area selected for further visual analysis. **(A_1-3_)** High magnification confocal images showing PV^+^ (white arrowhead), GABA^+^ (white arrow), and double-labeled PV^+^/GABA^+^ (white circle) neurons imaged from the location shown in the white box in **(A)**. Scale bar: 20 μm. **(B)** Representative traces of a single PV^+^/GABA^+^ neuron recorded from sSC (inset, yellow star). The white (rap; rapidly inactivating), black (non; non-spiking), and gray (fast; fast spiking) circles in the inset depict the location and corresponding firing pattern for each of the recorded neurons. **(C)** Proportion of PV^+^/GABA^+^ neurons according to their firing patterns from sSC and iSC. **(D)** Drawings of PV^+^/GABA^+^ neurons; **(D_1_)** horizontal, **(D_2_)** narrow field vertical, **(D_3_)** stellate in sSC, and **(D_4_)** stellate in iSC. Scale bar: 20 μm. **(D_5_)** Shows the locations of the neurons shown in **(D_1-4_)**. Note that all these neurons are portrayed in the inset in **(B)** and were rapidly inactivating (white circles).

**Figure 5A** shows a low magnification confocal image of a slice obtained from an Ai9;PV-Cre;GAD67 mouse showing the distributions of PV^+^ and GABA^+^ neurons in the SC. The distribution of PV^+^ (red, **Figure 5A_1_**), GABA^+^ (green, **Figure 5A_2_**), as well as PV^+^/GABA^+^ neurons (yellow, **Figure 5A_3_**), were similar to those observed in the Ai9;PV-Cre mouse treated with antibody against GABA (cf., **Figures 1D**, **5A**). The proportion of double-labeled PV^+^/GABA^+^ neurons in the Ai9;PV-Cre;GAD67 was similar to that found in the Ai9;PV-Cre mouse double-labeled with GABA antibody (sSC = 43 ± 4%; iSC = 73 ± 7%; Cx = 98 ± 1%; cf., **Supplementary Figure S3A** and **Figure 2B**; *p* = 0.71, *p* = 0.82, *p* = 0.26, respectively; *t*-test). **Figures 5A**_**1–3**_ display high magnification confocal images from the region highlighted by the white square in **Figure 5A** and show the presence of PV^+^ (arrowhead), GABA^+^ (arrows) and PV^+^/GABA^+^ neurons (circles) in the sSC. We obtained whole-cell recordings from 30, visually identified PV^+^/GABA^+^ neurons from both collicular layers, many of which we filled with biocytin. Surprisingly, most PV^+^/GABA^+^ neurons tended to display rapidly inactivating spiking patterns, as illustrated in the recording trace from a sSC neuron (**Figure 5B**; yellow star, inset), and they were found throughout the mediolateral and dorsoventral extent of both SC layers (**Figure 5B**, inset, circles). Quantitative analysis revealed that 88% of the PV^+^/GABA^+^ neurons in sSC and 64% in the iSC showed rapidly inactivating firing (**Figure 5B** inset, white circles; **Figure 5C**, white bars, rap). The other 12% in the sSC showed non-spiking profiles in response to positive currents steps (500 ms, -100 to +700 pA; **Figure 5B** inset, black circles, **Figure 5C**, black bars, non). In the iSC, 14% were non-spiking and 21% fast-spiking (**Figure 5B** inset, gray circles and **Figure 5C**, gray bars, fast). The physiological differences between PV^+^ and PV^+^/GABA^+^ neurons did not result from a systematic bias in recording locations between mouse lines, since the distributions of recorded neurons were found throughout the SC (**Supplementary Figure S3** and **Figure 5B**, inset). It seems likely that PV^+^/GABA^+^ neurons are smaller on average than PV^+^ neurons, and so these rapidly inactivating neurons were sampled less frequently (**Figure 3D**). Consistent with this idea, we found that the mean total resistance of PV^+^ and PV^+^/GABA^+^ neurons as measured from the membrane test window of the *Clampfit 10* software, were statistically different (286.1 ± 19 MΩ; 527 ± 54 MΩ, respectively; *p* < 0.05 paired *t*-test). Our visual inspection of the neurons during recordings as well as in confocal imaging, corroborated the hypothesis that PV^+^/GABA^+^ neurons were on average smaller than PV^+^ neurons overall. Based on these results, we conclude that most PV^+^/GABA^+^ neurons exhibit a unique physiological phenotype in both the sSC and iSC; namely, a rapidly inactivating firing pattern. As we found with PV^+^ neurons, PV^+^/GABA^+^ neurons showed a variety of different morphologies. **Figures 5D_1–4_** show examples of filled PV^+^/GABA^+^ neurons and **Figure 5D_5_** indicates the locations of these neurons within the SC. PV^+^/GABA^+^ neurons displayed horizontal (**Figure 5D_1_**), narrow field vertical (**Figure 5D_2_**), and stellate morphologies (**Figure 5D_3_**) in sSC, and radial-stellate morphology in iSC (**Figure 5D_4_**). The variety of PV^+^/GABA^+^ neuronal morphologies observed is similar to that observed in PV^+^ neurons, even though most neurons showed the rapidly inactivating physiological profile. Thus, PV^+^/GABA^+^ neurons in the SC constitute a particular neuronal subclass with heterogeneous morphology and rapidly inactivating spiking features, a considerably different profile than PV^+^ neurons in cerebral cortex, hippocampus and striatum.

### PV^+^/GABA^+^ Neurons in SC Make Functional GABAergic Synapses

**FIGURE 6:**
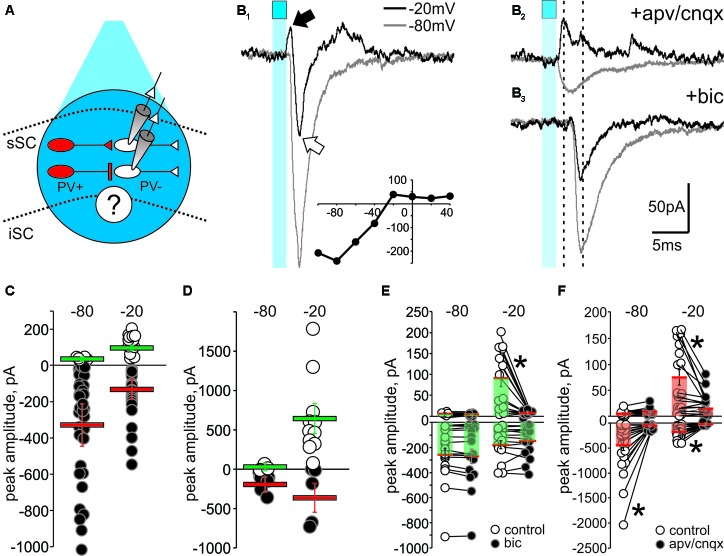
Optogenetic activation of PV^+^ neurons reveals functional GABAergic synapses in the SC. **(A)** Schematic diagram depicting the experimental design. Whole-cell recordings from PV^-^ neurons (white) were obtained from visually identified neurons using IR-DIC in slices from Ai32;PV-Cre mice. To stimulate PV^+^ neurons (red), pulses of blue light from a LED source were triggered through the objective (cyan circle, ∼5–10 mW). **(B_1_)** 2 ms pulses of LED stimulation (cyan square) elicited inward currents when the holding voltage (*V*_h_) of PV^-^ neurons was set at –80 mV (gray traces). With *V*_h_ set at –20 mV (black traces), light stimulation evoked a biphasic response, with an outward current (black arrow) preceding an inward current (white arrow). Inset, I/V curve. **(B_2_)** Bath application of APV/CNQX (50 μM/10 μM) diminished the light-evoked EPSC at *V*_h_ –80 mV and –20 mV and isolated the light-evoked outward current at –20 mV (black). **(B_3_**) After washout, bath application of bicuculline (10 μM) eliminated the IPSC at *V*_h_ –20 mV (black), thus isolating the EPSCs. **(C,D)** Peak amplitude plotted for all recorded IPSCs (white circles) and EPSCs (black circles) evoked in PV^-^ neurons by optogenetic activation of PV+ neurons in sSC **(C)** and in Cx **(D)**. Red bars: EPSC mean ± SE; green bars: IPSC mean ± SE. **(E)** Peak amplitudes of light-evoked current measured in PV^-^ neurons of sSC before (o) and after (∙) bath application of bicuculline, which exclusively inhibited the light-induced outward currents recorded in PV^-^ neurons at –20 mV. ^∗^*p* < 0.05, paired *t*-test. **(F)** Peak amplitude currents before (○) and after (●) bath application of APV/CNQX.

We next asked whether PV^+^ neurons made functional GABAergic synapses with PV^-^ neurons in the SC. To do this, we crossed Ai32 and PV-IRES-Cre mice to express ChR2-EYFP specifically in PV^+^ neurons. We obtained brain slices from this crossed mice (Ai32;PV-Cre) and recorded from collicular PV^-^ neurons (**Figure 6A**, white), while stimulating PV^+^ neurons (**Figure 6A**, red). Pulses of blue light (2 ms, 470 nM) produced sufficient membrane depolarization to evoke action potentials in PV^+^ neurons, indicating effective activation of ChR2 (**Supplementary Figure S4A**). This stimulation generated reliable post-synaptic currents (PSCs) in sSC PV^-^ neurons. We were unable to record PSCs from iSC PV^-^ neurons, in spite of effective PV^+^ neuronal stimulation (**Supplementary Figure S5**). The lack of postsynaptic responses in the iSC with PV^+^ ChR2 activation may be due to the scarcity of iSC PV^+^ neurons, thus rendering little and/or weak postsynaptic activity within this layer. An alternative, not mutually exclusive explanation is that few or no iSC PV^+^ neurons project within the layer, and that they instead project mostly to regions outside the SC. Or less likely, that we failed to record from neurons receiving PV^+^ input. Note however, that the lack of inhibitory responses in iSC with PV^+^ stimulation, suggests that local inhibition within the iSC (Helms et al., 2004; Lee and Hall, 2006; Lee et al., 2007) is mediated by circuits other than those involving PV^+^ neurons, such as somatostatin interneurons. Since we did not drive iSC neurons postsynaptically, all of the postsynaptic activation data come from recordings in the sSC.

**Figure 6B_1_** shows that 2 ms light stimulation (blue squares) evoked a biphasic response in a PV^-^ neuron when the holding voltage (V_h_) was -20 mV; an outward current followed by a large inward current (**Figure 6B_1_**, black trace). Similar stimulation resulted in a large inward current when *V*_h_ was -80 mV (**Figure 6B_1_**, gray trace). We plotted the I/V curve obtained by measuring the peaks of postsynaptic currents evoked in PV^-^ neurons held at different *V*_h_ (-100 to +40 mV) in response to light activation of PV^+^ neurons (**Figure 6B_1_**, inset). To investigate the neurotransmitters involved in these currents, we applied the glutamate receptor antagonists D-(-)-2-amino-5-phosphonopentanoic acid (APV) and 6-cyano-7-nitroquinoxaline-2,3-dione (CNQX). Bath application of these antagonists resulted in loss of the light-activated inward currents seen at both -80 mV and -20 mV, demonstrating the glutamatergic nature of the inward currents and isolating a PV^+^ driven outward current at -20 mV (**Figure 6B_2_**, top). After washout (**Supplementary Figure S4B**), subsequent application of the GABA_A_ receptor antagonist bicuculline eliminated the outward current (**Figure 6B_3_**), confirming its GABAergic origin.

Quantification of the EPSCs and IPSCs evoked by PV^+^ stimulation across our sample of 45 neurons revealed inward currents seen with *V*_h_ at -80 mV. About 95% of the recorded PV^-^ neurons showed inward currents with a mean amplitude of -355 ± 67 pA (**Figure 6C**, black circles and red bar), whereas only 10% of the neurons showed outward currents at -80 mV with a mean amplitude of 5.41 ± 0.66 pA (**Figure 6C**, white circles and green bar). With *V*_h_ set to -20 mV, closer to the reversal potential for Na^2+^, light pulses were more likely to evoke IPSCs. In fact, 73% of the PV^-^ neurons showed IPSCs with a mean amplitude of 64 ± 11 pA (**Figure 6C**, white circles and green bar). For comparison, and to confirm the efficacy of the ChR2 expression in PV^+^ neurons, we also performed similar recordings in cortex. As expected, light activation predominantly resulted in large amplitude IPSCs (mean = 639 ± 152 pA) at *V*_h_ -20 mV (**Figure 6D**).

To directly test whether activation of ChR2 in PV^+^ neurons triggers GABAergic or glutamatergic synapses, we bath applied bicuculline or APV/CNQX and measured the amplitude of the evoked PSCs before and after bath application. **Figure 6E** shows that bicuculline failed to reduce the EPSC amplitude recorded at either -80 mV (control: -258 ± 53 pA; bic: -269 ± 56 pA; *p* = 0.18) or -20 mV (control: -178 ± 31 pA; bic: -150 ± 33 pA; *p* = 0.33), but significantly reduced the IPSCs observed at -20 mV (control: 90.49 ± 19.84 pA; bic: 2.96 ± 1.29 pA; ^∗^*p* < 0.05), demonstrating the GABAergic nature of these synapses. Most IPSCs recorded at -80 mV were small and not affected by bicuculline (control: 3.92 ± 0.39 pA; bic; 3.55 ± 0.50 pA, *p* = 0.58). On the other hand, bath application of APV/CNQX significantly reduced the EPSCs at both -80 mV (control: -447 ± 100 pA; apv/cnqx: -43 ± 9 pA; ^∗^*p* < 0.05) and -20 mV (control: -173 ± 29 pA; apv: -21 ± 2 pA; ^∗^*p* < 0.05; **Figure 6F**), supporting the idea that these inward currents are mediated by glutamatergic receptors. We also found that some of the outward currents recorded at -20 mV were significantly inhibited by APV/CNQX, suggesting that glutamatergic PV^+^ synapses in the SC are also driving some of the IPSCs (control: 74 ± 15; apv: 14 ± 4, *p* < 0.05). Taken together, these results provide evidence for the existence of both PV^+^ GABAergic and PV^+^ glutamatergic synaptic transmission capable of driving inhibitory circuits within the SC.

### PV^+^ Neurons Form Direct Inhibitory and Feedforward Inhibitory Circuits in SC

**FIGURE 7:**
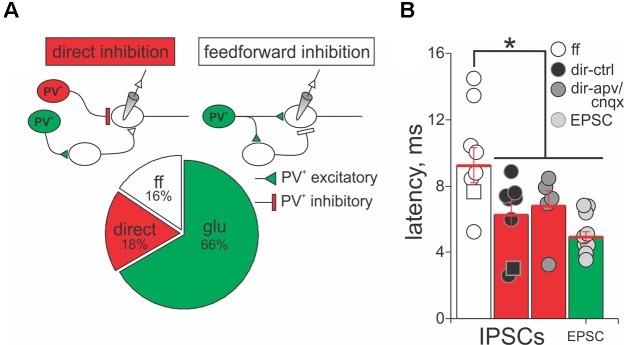
PV^+^ neurons form direct and feedforward inhibitory circuits in the SC. **(A)** Top panel, schematic diagrams of the PV^+^ driven inhibitory circuits, direct and feedforward, found in our recordings. PV^+^, red circles; PV^-^, white circles. Triangles depict excitatory terminals and bars depict inhibitory terminals. Lower panel, percentage of recordings providing evidence for direct inhibition (18%), feedforward inhibition (16%), and excitatory (66%) circuits resulting from optogenetic activation of PV^+^ neurons. **(B)** Latency of the peak outward currents classified as feedforward (ff, white circles), direct inhibition before (dir-ctrl, black circles), and after APV/CNQX application (dir-apv/cnqx, gray circles). The latency of the peak inward currents in neurons showing feedforward inhibition is also shown (EPSC, light gray circles). Red bar: mean ± SE.

The results shown in **Figure 6** suggest that PV^+^ neurons in the SC form excitatory and inhibitory synaptic connections with PV^-^ neurons, consistent with our anatomical results showing that some, but not all PV^+^ neurons in the SC co-localize GABA. Therefore, we quantified the nature and kinetics of light-evoked PSCs in the presence of APV/CNQX or bicuculline for our sample of 45 PV^-^ neurons recorded from the Ai32;PV-Cre mouse. The upper panels in **Figure 7A** show schematic drawings of hypothetical synaptic circuits that could explain the outward currents obtained in response to optogenetic stimulation of PV^+^ neurons, as shown in **Figure 6B** and **Supplementary Figure S4C**. The IPSCs were classified as feedforward inhibition if bath application of APV/CNQX abolished the light-evoked outward current or as direct inhibition if APV/CNQX failed to abolish the outward current. Again, to corroborate their GABAergic nature, after washout, bath application of bicuculline eliminated the outward currents in all neurons displaying feedforward and direct inhibitory currents. The pie chart in **Figure 7A** shows that 34% of our recordings showed PV^+^ neurons driving inhibitory circuits; of these 18% corresponded to APV/CNQX-insensitive outward currents, inhibited by bicuculline, and thus resulted from PV^+^ driven direct inhibitory synapses. The rest, 16% corresponded to APV/CNQX sensitive outward currents, consistent with feedforward inhibitory circuity. From our recordings, 66% showed APV/CNQX-sensitive inward currents, consistent with direct excitation via glutamatergic synapses.

A signature feature of feedforward inhibitory circuits is that IPSCs are recorded later compared to directly activated inward currents. Therefore, we measured the latency of isolated currents as the times from the beginning of the light pulse to the peak of the evoked PSC, to assess whether they were well explained by direct or feedforward circuits. **Figure 7B** shows the mean latency of the peak amplitude of IPSCs and EPSCs recorded from PV^-^ neurons in response to light activation of PV^+^ neurons, when sorted by whether the currents were classified as direct or feedforward inhibition, according to their APV/CNQX-sensitivity. For the neurons showing feedforward inhibitory profile, the mean latency of the peak outward current was 9.25 ms ± 1.07 ms with a minimum of 5.24 ms and a maximum of 14.49 ms (white circles and bar). The mean value was significantly longer than the mean latency calculated for the currents classified as direct inhibition (6.24 ms ± 0.80 ms with a minimum of 2.59 ms and a maximum of 14.49 ms, black circles and bars; Mann–Whitney *p* = 0.032), as well as for the EPSCs (4.08 ms ± 0.32 ms with a minimum of 3.00 ms and a maximum of 5.88 ms; light gray circles and bars; Mann–Whitney *p* = 0.002). This longer latency outward current is therefore consistent with a feedforward mechanism. In addition, the mean latency of direct inhibitory and excitatory currents (6.24 ms versus 4.08 ms) did not differ significantly (**Figure 7B**, cf., black and light gray circles and bars, Mann–Whitney *p* = 0.83). To ensure that the inward currents did not confound the measurements of the outward currents, we measured the latency of direct outward currents before and after application of APV/CNQX and found that these values showed no statistical differences (cf., black and gray circles and bars, Mann–Whitney *p* = 0.84). These results demonstrate that PV^+^ neurons can form excitatory, as well as two types of inhibitory, circuits with PV^-^ neurons, with direct and feedforward inhibition, occurring in equal proportions.

## Discussion

We took advantage of a mouse line genetically engineered to express the reporter gene (tdTomato) in PV^+^ neurons to determine their distribution and electrophysiological properties within the SC. Consistent with previous reports, we found strong PV expression concentrated in a discrete sublamina of the visuosensory layer (sSC) and weaker PV^+^ expression, with a patch-like appearance, in the motor layer (iSC) (Illing et al., 1990; Mize et al., 1992; Cork et al., 1998; Jeong et al., 2014; Lee et al., 2014). While PV marks a very specific subclass of GABAergic fast-spiking inhibitory interneurons in the cerebral cortex, striatum and hippocampus (Hu et al., 2014), our results showed that PV^+^ neurons in the sSC present heterogeneous spiking profiles and morphologies, and only a fraction contain GABA. On the other hand, a higher proportion of the relatively sparse PV^+^ population in the iSC showed the presence of GABA. Furthermore, most of these neurons showed a fast-spiking profile and radial-stellate morphology. Crossing of the GAD67-EGFP mouse with the Ai9;PV-Cre mouse to identify PV^+^/GABA^+^ neurons in the SC confirmed our immunohistological results showing that in the iSC, a larger proportion of PV^+^ neurons express GABA, compared to the sSC. Surprisingly, the majority of PV^+^/GABA^+^ neurons, in both SC layers, showed rapidly inactivating spiking patterns, an entirely different profile from that seen in cortex, striatum or hippocampus. Finally, activation of PV^+^ neurons expressing ChR2 revealed three main types of connections with PV^-^ neurons in the SC, direct excitatory, direct inhibitory and feedforward inhibitory synaptic connections. Taken together, our immunohistological, electrophysiological and optogenetic results indicate that the PV^+^ subclass of neuron plays multiple roles in SC circuits, and these roles are most likely different from those played by PV^+^ neurons in cortical, striatal or hippocampal areas (Gray et al., 2014; Chen et al., 2017).

### Comparison to Previous Findings in the SC Superficial Layers (sSC)

The pattern of PV staining observed in the transgenic Ai9;PV-Cre mouse sSC (**Figures 1**, **2**) was similar to that observed previously using immunohistochemistry (Illing et al., 1990; Mize et al., 1992; Cork et al., 1998; González-Soriano et al., 2000; Behan et al., 2002; Kang et al., 2002), with a predominant band of labeled neurons in the lower SGS, and others scattered across the sSC. Interestingly, this band pattern is not obvious in the monkey sSC, where the distribution appears homogeneous (Soares et al., 2001).

An outstanding feature of sSC PV^+^ neurons, especially when compared with their cortical homologs, is the degree of heterogeneity of their physiological properties. Within this population, we found 5 different spiking classes, as previously described (Lee et al., 2001; Edwards et al., 2002). We observed that these classes appear to sort independently of their morphology, in agreement with previous *in vitro* studies of sSC neurons (Edwards et al., 2002). Others have suggested that some intrinsic biophysical properties are correlated to specific neuronal type (Gale and Murphy, 2014), however, our data do not support this idea. Previous studies have suggested the existence of a link between cellular Ca^2+^-binding protein expression and neuronal excitability (Celio, 1986; Gall et al., 2003; Roussel et al., 2006). In fact, theoretical work suggests that the excitability of fast-spiking neurons in the striatum depends on PV concentration and the presence of Ca^2+^-activated K^+^ channels, such as SK channels (Bischop et al., 2012). In contrast, our results show that sSC PV^+^ neurons display a variety of spiking profiles and that PV^+^/GABA^+^ neurons show a very consistent, rapidly inactivating spiking profile. Possible explanations for these differences include; SC PV^+^ neurons express different kinetic relationships between PV and SK channels, they have other Ca^2+^-activated K^+^ channels that allow them to buffer Ca^2+^ at different concentrations or they have Ca^2+^-activated K^+^ channels with slower kinetics.

Rapidly inactivating PV^+^/GABA^+^ neurons would provide a fast, transient inhibitory input to their targets in response to retinal stimulation. It seems reasonable to propose that non-spiking activity might be found in inhibitory neurons that lack axons and instead, produce inhibition using dendrodendritic synapses, similar to retinal horizontal cells. Dendrodendritic synapses are known to modulate retinal inputs in the sSC (Mize et al., 1982; Mize, 1992). In this case, the inhibitory inputs would arrive soon after retinal excitation, in a tonic fashion, like that provided by fast-spiking neurons. Together, these signals would cause the sSC PV^+^/GABA^+^ neurons to function more as event detectors, looking for transient changes in the visual scene (Kaneda and Isa, 2013). More recent experiments, using transgenic and knock-in mice, assessed the features of sSC neurons containing glutamic acid decarboxylase (GAD; GAD67-GFP or Gad2-Cre mice). These studies indicate that only horizontal neurons are positive for GAD (Endo et al., 2003; Gale and Murphy, 2014). Since the stellate and narrow field vertical neurons we observed were not labeled in these studies, it suggests that the GAD mice reveal only a subset of the inhibitory neurons present in the sSC (Whyland et al., 2017).

### Comparison to Previous Findings in the Motor Layers (iSC)

Parvalbumin expression in the iSC is densest laterally, and appears patchy medio-laterally, similar to that descriptions using immunohistochemistry and may be due to the presence of PV^+^ terminal puffs (Illing et al., 1990; Mize et al., 1992; Cork et al., 1998; González-Soriano et al., 2000; Behan et al., 2002). PV^+^ neurons in iSC displayed more homogeneity compared to sSC, with most neurons showing a fast-spiking profile and radial-stellate morphology. These findings mirror immunohistochemical descriptions of iSC PV^+^ neurons in other species, although the terms used to describe these neurons vary (Mize et al., 1992; González-Soriano et al., 2000). There is evidence that many PV^+^ multipolar neurons supply the crossed tectoreticular and tectotectal projections (Mize, 1992). The tectotectal projection is known to contain a strong inhibitory component in the cat (Takahashi et al., 2005, 2010). It seems likely then, that the radial-stellate neurons observed in the present paper are similar to the multipolar neurons others have observed supplying the tectotectal projection (Rhoades et al., 1986; Olivier et al., 1998). It would be quite surprising if the crossed tectoreticular neurons supplying the paramedian pontine reticular formation were GABAergic. However, others have suggested that GABAergic neurons in the iSC do have targets outside the colliculus, as well as ascending interlaminar projections (Sooksawate et al., 2011). Our difficulty in recording IPSCs in iSC PV^-^ neurons after ChR2 activation of PV^+^ neurons is consistent with the possibility that they are projection cells, not local circuit neurons.

### PV^+^ Circuit Motifs in the Visuosensory SC

Our immunohistochemical and optogenetic experiments provide important constraints on sSC circuit models. Although PV is commonly associated with GABAergic neurons, in the sSC it does not appear to be an exclusive marker of inhibitory neurons nor of a particular electrophysiological phenotype, as it does in cortex, striatum or hippocampus. Rather, PV may be a marker for a functional circuit that includes both excitatory and inhibitory neurons. It has been proposed that PV^+^ neurons integrate and transmit Y-type retinal ganglion cell information and cortical Y-like information to thalamic nuclei and to the pontine gray (Casagrande, 1994; Mize, 1996). In this model, PV^+^ neurons are exclusively excitatory. Similarly, recent work using transgenic mice showed an excitatory circuit from the sSC to the amygdala via the parabigeminal nucleus (Shang et al., 2015). Our results are largely consistent with those of Shang et al. (2015), but extend them in important ways. Our optogenetic experiments suggest that PV^+^ neurons form at least three types of intrinsic circuits with sSC PV^-^ neurons: direct excitation, direct inhibition and feedforward inhibition. It will be interesting to determine whether these circuits arise from different neuronal cell types. For example, the direct excitatory projection may emanate from the axon collaterals of glutaminergic narrow field vertical neurons (Hall and Lee, 1997), the GABAergic, direct inhibitory projection may originate from the horizontal and stellate neurons, and the feedforward inhibitory projection may originate from intrinsic circuits or activation of retinal ganglion cell axons terminating on a PV^-^ subclass of inhibitory interneuron (Schmidt et al., 2001; Edwards and Platt, 2003).

We believe that intrinsic PV^+^/GABA^+^ circuits, rather than external sources mediate the feedforward inhibitory currents seen in the sSC for the following reasons. First, activation of retinal axons generally results in triphasic responses in target neurons with an initial inward current, followed by an outward current and a third inward current, suggestive of recurrent excitation (Isa et al., 1998). Our results show that ChR2 activation in PV^+^ neurons evoked biphasic currents, indicative of intrinsic feedforward inhibition. Second, we do not believe the direct inhibitory currents we measured resulted from activation of external sources of GABA, such as the substantia nigra pars reticulata, whose neurons co-localize PV^+^ (White et al., 1994; Lee and Tepper, 2007). Although our current results do not allow us to rule this out definitively, we think that activation of nigral afferents is an unlikely explanation due to the small size of the inhibitory currents we measured compared to the large-sized potentials that stimulation of the nigra evokes in SC neurons (Karabelas and Moschovakis, 1985). Furthermore, in all species so far examined, the input from the nigra to the sSC is less prominent than that to the motor layers (Harting et al., 2001). It is noteworthy that, in our model, activation of PV^+^/GABA^+^ neurons from the substantia nigra pars reticulata (SNr) did not elicit inhibitory influence over collicular neurons. Ongoing experiments in which viral injections carrying ChR2 that target the SNr will help to further elucidate the role of SNr activation on the collicular circuitry.

## Conclusion

We provide novel evidence that some PV^+^ neurons in the SC co-localize GABA in both the visuosensory and motor layers, with the latter being more prominent. PV^+^ neurons in the sSC have heterogeneous morphology and firing properties allowing them to respond selectively to different inputs and support varied functions, whereas most PV^+^ neurons in the iSC show fast-spiking activity and radial-stellate morphology. Unlike PV^+^ neurons elsewhere in the brain, most SC neurons that co-localize PV and GABA displayed rapidly inactivating firing properties. Furthermore, these neurons form both feedforward and direct inhibitory circuits within the SC. These properties indicate that PV^+^ neurons in the SC perform unique circuit functions that are different from the canonical roles described in other areas of the brain, and may be specialized for different functions in the different layers of the SC.

## Author Contributions

CV, PL, PM, and MB designed the experiments. CV and QW collected the data. CV, QW, PL, and PM analyzed the data. CV wrote the first draft of the manuscript. CV, PL, PM, and MB contributed to manuscript revision, read, and approved the submitted version.

## Conflict of Interest Statement

The authors declare that the research was conducted in the absence of any commercial or financial relationships that could be construed as a potential conflict of interest.

## Supplementary Material

FIGURE S1 Histological sections through the sSC **(A)** and iSC **(B)** of an Ai9;PV-Cre mouse. Cells labeled with Neurotrace^®^ appear in green (pseudo-colored) and PV^+^ cells (with tdTomato, red) appear as yellow. All PV^+^ cells co-localized with Neurotrace^®^ indicating they are neurons.

FIGURE S2 Distribution of PV^+^ neurons in the SC layers recorded in brain slices obtained from the Ai9;PV-Cre mouse. Each circle corresponds to individually recorded PV+ neurons and the color shows their respective electrophysiological phenotype (gray, fast spiking; white; rapidly inactivating; black, regular spiking).

FIGURE S3 The percentage of PV^+^/GABA^+^ neurons from the total number of PV^+^ neurons in the Ai9;PV-Cre;GAD67 mouse are similar to those found in the Ai9;PV-Cre mouse using a GABA antibody. **(A)** Mean percentage of PV^+^/GABA^+^ vs. PV^+^ neurons calculated from slices obtained from the Ai9;PV-Cre mouse using a GABA antibody (white bars, **Figure 2B**) compared to the mean percentage of PV^+^/GABA^+^ neurons found in three Ai9;PV-Cre;GAD67 mice (gray bars); sSC = 42 ± 4% (black circles), iSC = 73 ± 7% (white circles), Cx = 98 ± 2%; *n* = 34 (gray circles). **(B)** PV^+^ (tdTomato), GABA^+^ (GFP) and merge panels showing high magnification confocal images from sections of sSC **(B_1_)**, iSC **(B_2_)**, and Cx **(B_3_)** obtained from an Ai9;PV-Cre;GAD67 mouse. Examples of double-labeled PV^+^/GABA^+^ neurons are highlighted on each of the images (white circles). Red bar: mean ± SE.

FIGURE S4 **(A)** Brief LED blue light pulses (cyan square, 2 ms) effectively evoked action potentials in PV^+^ neurons recorded in slices from Ai32;PV-Cre mice. Representative trace of a PV^+^ neuron (red trace) from sSC recorded in current-clamp configuration without QX-314 in the pipette (dashed lines, 0 mV and -60 mV). **(B)** Light-evoked EPSCs recorded at -80 mV (black trace) were blocked by APV/CNQX (50 μM/10 μM) (green trace). After ∼15 min washout, the EPSC amplitude was almost completely recovered (red trace). Bicuculline application (10 μM) did not affect the EPSC amplitude (orange trace). **(C)** Representative traces showing a PV^+^ driven inhibitory circuits classified as feedforward inhibition. Bath application of APV/CNQX diminished PSCs evoked at -80 and -20 mV. After washout, bicuculline selectively inhibited the outward current. Scale: 100 pA/5 ms.

FIGURE S5 Optogenetic stimulation of PV^+^ neurons failed to evoke PSCs in PV^-^ neurons recorded in iSC. **(A)** Fluorescent image (40×, GFP) showing a PV^+^ neuron in iSC (white arrow) of a brain slice from the Ai32;PV-Cre mouse. Scale: 20 μm. **(B)** DIC image showing the location of the recorded PV^+^ neuron. Scale: 200 μm. **(C)** Upper panel: Schematic illustration showing the recording of PV^-^ (1) and PV^+^ (2) neurons in the iSC. Bottom panel: Representative traces of a recorded PV^-^ neuron showing no significant current evoked upon 2 ms light stimulation in voltage-clamp configuration. Note the spontaneous IPSC evoked long after the light pulse when voltage is held at -20 mV (white arrow). Scale: 10 pA/5 ms. **(D)** Light stimulation elicited in a PV^+^ neuron a large and short latency APV/CNQX-insensitive inward current at -80 mV. Application of 1 μM TTX eliminated the inward current. The recording pipette contained QX-314 hence the absence of spikes. Scale: 100 pA/5 ms. **(E)** Light pulses of increasing length failed to evoke PSCs in iSC PV^-^ neurons at either -80 mV or -20 mV *V*_m_. **(F)** a train of 10 light pulses (5 ms in length) failed to evoke PSCs in iSC PV^-^ neurons. Five consecutive traces are superimposed.

## References

[B1] Basso M. A. May P. J. (2017). Circuits for action and cognition: a view from the superior colliculus. 3 197–226. 10.1146/annurev-vision-102016-061234 28617660 PMC5752317

[B2] Behan M. Jourdain A. Bray G. M. (1992). Calcium binding protein (calbindin D28k) immunoreactivity in the hamster superior colliculus: ultrastructure and lack of co-localization with GABA. 89 115–124. 10.1007/BF00229008 1601089

[B3] Behan M. Steinhacker K. Jeffrey-Borger S. Meredith M. A. (2002). Chemoarchitecture of GABAergic neurons in the ferret superior colliculus. 452 334–359. 10.1002/cne.10378 12355417

[B4] Bischop D. P. Orduz D. Lambot L. Schiffmann S. N. Gall D. (2012). Control of neuronal excitability by calcium binding proteins: a new mathematical model for striatal fast-spiking interneurons. 5:78. 10.3389/fnmol.2012.00078 22787441 PMC3392946

[B5] Cardin J. A. Carlén M. Meletis K. Knoblich U. Zhang F. Deisseroth K. (2009). Driving fast-spiking cells induces gamma rhythm and controls sensory responses. 459 663–667. 10.1038/nature08002 19396156 PMC3655711

[B6] Casagrande V. A. (1994). A third parallel visual pathway to primate area V1. 17 305–310. 10.1016/0166-2236(94)90065-5 7524217

[B7] Cauli B. Audinat E. Lambolez B. Angulo M. C. Ropert N. Tsuzuki K. (1997). Molecular and physiological diversity of cortical nonpyramidal cells. 17 3894–3906. 10.1523/JNEUROSCI.17-10-03894.1997 9133407 PMC6573690

[B8] Celio M. R. (1986). Parvalbumin in most y-aminobutyric acid-containing neurons of the rat cerebral cortex. 231 995–997. 10.1126/science.3945815 3945815

[B9] Chattopadhyaya B. (2004). Experience and activity-dependent maturation of perisomatic GABAergic innervation in primary visual cortex during a postnatal critical period. 24 9598–9611. 10.1523/JNEUROSCI.1851-04.2004 15509747 PMC6730138

[B10] Chen G. Zhang Y. Li X. Zhao X. Ye Q. Lin Y. (2017). Distinct inhibitory circuits orchestrate cortical beta and gamma band oscillations. 96 1403.e6–1418.e6. 10.1016/j.neuron.2017.11.033 29268099 PMC5864125

[B11] Chung D. W. Fish K. N. Lewis D. A. (2016). Pathological basis for deficient excitatory drive to cortical parvalbumin interneurons in schizophrenia. 173 1131–1139. 10.1176/appi.ajp.2016.16010025 27444795 PMC5089927

[B12] Cork R. J. Baber S. Z. Mize R. R. (1998). Calbindin(D28k)- and parvalbumin-immunoreactive neurons form complementary sublaminae in the rat superior colliculus. 394 205–217. 10.1002/(SICI)1096-9861(19980504)394:2<205::AID-CNE5>3.0.CO;2-6 9552126

[B13] Edwards M. D. Platt B. (2003). Sometimes you see them, sometimes you don’t: IPSCs in the rat superficial superior colliculus. 149 331–339. 10.1007/s00221-002-1368-2 12632235

[B14] Edwards M. D. White A. M. Platt B. (2002). Characterisation of rat superficial superior colliculus neurones: firing properties and sensitivity to GABA. 110 93–104. 10.1016/S0306-4522(01)00558-9 11882375

[B15] Endo T. Yanagawa Y. Obata K. Isa T. (2003). Characteristics of GABAergic neurons in the superficial superior colliculus in mice. 346 81–84. 10.1016/S0304-3940(03)00570-6 12850553

[B16] Gale S. D. Murphy G. J. (2014). Distinct representation and distribution of visual information by specific cell types in mouse superficial superior colliculus. 34 13458–13471. 10.1523/JNEUROSCI.2768-14.2014 25274823 PMC4180477

[B17] Gall D. Roussel C. Susa I. D’Angelo E. Rossi P. Bearzatto B. (2003). Altered neuronal excitability in cerebellar granule cells of mice lacking calretinin. 23 9320–9327. 10.1523/JNEUROSCI.23-28-09320.2003 14561859 PMC6740583

[B18] Gonchar Y. Wang Q. Burkhalter A. (2008). Multiple distinct subtypes of GABAergic neurons in mouse visual cortex identified by triple immunostaining. 1:3. 10.3389/neuro.05.003.2007 18958197 PMC2525923

[B19] González-Soriano J. González-Flores M. L. Contreras-Rodríguez J. Rodríguez-Veiga E. Martínez-Sainz P. (2000). Calbindin D28k and parvalbumin immunoreactivity in the rabbit superior colliculus: an anatomical study. 259 334–346. 10.1002/1097-0185(20000701)259:3<334::AID-AR100>3.0.CO;2-K 10861366

[B20] Gray D. T. Engle J. R. Rudolph M. L. Recanzone G. H. (2014). Regional and age-related differences in GAD67 expression of parvalbumin- and calbindin-expressing neurons in the rhesus macaque auditory midbrain and brainstem. 522 4074–4084. 10.1002/cne.23659 25091320 PMC4263274

[B21] Gupta A. (2000). Organizing principles for a diversity of GABAergic interneurons and synapses in the neocortex. 287 273–278. 10.1126/science.287.5451.273 10634775

[B22] Hall W. C. Lee P. (1997). Interlaminar connections of the superior colliculus in the tree shrew. III: the optic layer. 14 647–661. 10.1017/S095252380001261X 9278994

[B23] Harting J. K. Updyke B. V. Van Lieshout D. P. (2001). The visual-oculomotor striatum of the cat: functional relationship to the superior colliculus. 136 138–142. 10.1007/s002210000606 11204409

[B24] Hashemi E. Ariza J. Rogers H. Noctor S. C. Martínez-Cerdeño V. (2017). The number of parvalbumin-expressing interneurons is decreased in the medial prefrontal cortex in autism. 27 1931–1943. 10.1093/cercor/bhw021 26922658 PMC6074948

[B25] Helms M. C. Özen G. Hall W. C. (2004). Organization of the intermediate gray layer of the superior colliculus. i. intrinsic vertical connections. 91 1706–1715. 10.1152/jn.00705.2003 15010497

[B26] Hu H. Gan J. Jonas P. (2014). Fast-spiking, parvalbumin+ GABAergic interneurons: from cellular design to microcircuit function. 345:1255263. 10.1126/science.1255263 25082707

[B27] Illing R.-B. Vogt D. M. Spatz W. B. (1990). Parvalbumin in rat superior colliculus. 120 197–200. 10.1016/0304-3940(90)90037-A 1963482

[B28] Isa T. Endo T. Saito Y. (1998). The visuo-motor pathway in the local circuit of the rat superior colliculus. 18 8496–8504. 10.1523/JNEUROSCI.18-20-08496.1998 9763492 PMC6792861

[B29] Jeong S.-J. Kim H.-H. Lee W.-S. Jeon C.-J. (2014). Immunocytochemical localization of calbindin D28K, calretinin, and parvalbumin in bat superior colliculus. 47 113–123. 10.1267/ahc.14004 25320408 PMC4164697

[B30] Kaneda K. Isa T. (2013). GABAergic mechanisms for shaping transient visual responses in the mouse superior colliculus. 235 129–140. 10.1016/j.neuroscience.2012.12.061 23337535

[B31] Kang Y. S. Park W. M. Lim J. K. Kim S. Y. Jeon C. J. (2002). Changes of calretinin, calbindin D28K and parvalbumin-immunoreactive neurons in the superficial layers of the hamster superior colliculus following monocular enucleation. 330 104–108. 10.1016/S0304-3940(02)00723-1 12213644

[B32] Karabelas A. B. Moschovakis A. K. (1985). Nigral inhibitory termination on efferent neurons of the superior colliculus: an intracellular horseradish peroxidase study in the cat. 239 309–329. 10.1002/cne.902390305 2995462

[B33] Kawaguchi Y. (1993). Physiological, morphological, and histochemical characterization of three classes of interneurons in rat neostriatum. 13 4908–4923. 10.1523/JNEUROSCI.13-11-04908.1993 7693897 PMC6576359

[B34] Kawaguchi Y. Katsumaru H. Kosaka T. Heizmann C. W. Hama K. (1987). Fast spiking cells in rat hippocampus (CA1 region) contain the calcium-binding protein parvalbumin. 416 369–374. 10.1016/0006-8993(87)90921-8 3304536

[B35] Kawaguchi Y. Kubota Y. (1993). Correlation of physiological subgroupings of nonpyramidal cells with parvalbumin- and calbindinD28k-immunoreactive neurons in layer V of rat frontal cortex. 70 387–396. 10.1152/jn.1993.70.1.387 8395585

[B36] Kawaguchi Y. Kubota Y. (1997). GABAergic cell subtypes and their synaptic connections in rat frontal cortex. 7 476–486. 10.1093/cercor/7.6.476 9276173

[B37] Kim J. Kim Y. Nakajima R. Shin A. Jeong M. Park A. H. (2017). Inhibitory basal ganglia inputs induce excitatory motor signals in the thalamus. 95 1181.e8–1196.e8. 10.1016/j.neuron.2017.08.028 28858620

[B38] Klausberger T. Somogyi P. (2008). Neuronal diversity and temporal dynamics: the unity of hippocampal circuit operations. 321 53–57. 10.1126/science.1149381 18599766 PMC4487503

[B39] Laemle L. K. (1981). A Golgi study of cellular morphology in the superficial layers of superior colliculus of man, Saimiri, and Macaca. 22 253–263. 7276538

[B40] Langer T. P. Lund R. D. (1974). The upper layers of the superior colliculus of the rat: a Golgi study. 158 405–435. 10.1002/cne.901580404 4615112

[B41] Lee C. R. Tepper J. M. (2007). Morphological and physiological properties of parvalbumin- and calretinin-containing γ-aminobutyric acidergic neurons in the substantia nigra. 500 958–972. 10.1002/cne.21220 17177263

[B42] Lee J.-Y. Choi J.-S. Ahn C.-H. Kim I.-S. Ha J.-H. Jeon C.-J. (2006). Calcium-binding protein calretinin immunoreactivity in the dog superior colliculus. 39 125–138. 10.1267/ahc.06008 17327899 PMC1698867

[B43] Lee J.-Y. Jeong S.-J. Jeon C.-J. (2014). Parvalbumin-Immunoreactive cells in the superior colliculus in dog: distribution, colocalization with GABA, and effect of monocular enucleation. 31 748–757. 10.2108/zs140073 25366158

[B44] Lee P. Hall W. C. (2006). An *in vitro* study of horizontal connections in the intermediate layer of the superior colliculus. 26 4763–4768. 10.1523/JNEUROSCI.0724-06.2006 16672648 PMC6674151

[B45] Lee P. H. Helms M. C. Augustine G. J. Hall W. C. (1997). Role of intrinsic synaptic circuitry in collicular sensorimotor integration. 94 13299–13304. 10.1073/pnas.94.24.13299 9371840 PMC24303

[B46] Lee P. H. Schmidt M. Hall W. C. (2001). Excitatory and inhibitory circuitry in the superficial gray layer of the superior colliculus. 21 8145–8153. 10.1523/JNEUROSCI.21-20-08145.2001 11588187 PMC6763849

[B47] Lee P. H. Sooksawate T. Yanagawa Y. Isa K. Isa T. Hall W. C. (2007). Identity of a pathway for saccadic suppression. 104 6824–6827. 10.1073/pnas.0701934104 17420449 PMC1849959

[B48] Leuba G. Saini K. (1996). Calcium-binding proteins immunoreactivity in the human subcortical and cortical visual structures. 13 997–1009. 10.1017/S0952523800007665 8961531

[B49] Lewis D. A. Curley A. A. Glausier J. R. Volk D. W. (2012). Cortical parvalbumin interneurons and cognitive dysfunction in schizophrenia. 35 57–67. 10.1016/j.tins.2011.10.004 22154068 PMC3253230

[B50] Luksch H. Cox K. Karten H. J. (1998). Bottlebrush dendritic endings and large dendritic fields: motion-detecting neurons in the tectofugal pathway. 396 399–414. 10.1002/(SICI)1096-9861(19980706)396:3<399::AID-CNE9>3.0.CO;2-Y 9624592

[B51] Ma T. P. Cheng H.-W. Czech J. A. Rafols J. A. (1990). Intermediate and deep layers of the macaque superior colliculus: A golgi study. 295 92–110. 10.1002/cne.902950109 1692855

[B52] Markram H. Toledo-Rodriguez M. Wang Y. Gupta A. Silberberg G. Wu C. (2004). Interneurons of the neocortical inhibitory system. 5 793–807. 10.1038/nrn1519 15378039

[B53] May P. J. (2006). The mammalian superior colliculus: Laminar structure and connections. 151 321–378. 10.1016/S0079-6123(05)51011-2 16221594

[B54] Mize R. R. (1992). The organization of GABAergic neurons in the mammalian superior colliculus. 90 219–248. 10.1016/S0079-6123(08)63616-X 1321459

[B55] Mize R. R. (1996). Neurochemical microcircuitry underlying visual and oculomotor function in the cat superior colliculus. 112 35–55. 10.1016/S0079-6123(08)63319-1 8979819

[B56] Mize R. R. (1999). Calbindin 28kD and parvalbumin immunoreactive neurons receive different patterns of synaptic input in the cat superior colliculus. 843 25–35. 10.1016/S0006-8993(99)01847-8 10528107

[B57] Mize R. R. Jeon C.-J. Butler G. D. Luo Q. Emson P. C. (1991). The calcium binding protein calbindin-D 28K reveals subpopulations of projection and interneurons in the cat superior colliculus. 307 417–436. 10.1002/cne.903070307 1713236

[B58] Mize R. R. Luo Q. Butler G. Jeon C.-J. Nabors B. (1992). The calcium binding proteins parvalbumin and calbindin-D 28K form complementary patterns in the cat superior colliculus. 320 243–256. 10.1002/cne.903200208 1619052

[B59] Mize R. R. Spencer R. F. Sterling P. (1982). Two types of GABA-accumulating neurons in the superficial gray layer of the cat superior colliculus. 206 180–192. 10.1002/cne.902060207 7085927

[B60] Mooney R. D. Klein B. G. Rhoades R. W. (1985). Correlations between the structural and functional characteristics of neurons in the superficial laminae and the hamster’s superior colliculus. 5 2989–3009. 10.1523/JNEUROSCI.05-11-02989.1985 PMC6565159 4056863

[B61] Moschovakis A. K. Karabelas A. B. (1985). Observations on the somatodendritic morphology and axonal trajectory of intracellularly HRP-Labeled efferent neurons located in the deeper layers of the superior colliculus of the cat. 239 276–308. 10.1002/cne.902390304 4044941

[B62] Moschovakis A. K. Karabelas A. B. Highstein S. M. (1988a). Structure-function relationships in the primate superior colliculus. I. Morphological classification of efferent neurons. 60 232–262. 10.1152/jn.1988.60.1.232 3404219

[B63] Moschovakis A. K. Karabelas A. B. Highstein S. M. (1988b). Structure-function relationships in the primate superior colliculus. II. Morphological identity of presaccadic neurons. 60 263–302. 3404220 10.1152/jn.1988.60.1.263

[B64] Norita M. (1980). Neurons and synaptic patterns in the deep layers of the superior colliculus of the cat. A Golgi and electron microscopic study. 190 29–48. 10.1002/cne.901900104 7381053

[B65] Olivier E. Porter J. D. May P. J. (1998). Comparison of the distribution and somatodendritic morphology of tectotectal neurons in the cat and monkey. 15 903–922. 10.1017/S095252389815513X 9764533

[B66] Rhoades R. W. Mooney R. D. Szczepanik A. M. Klein B. G. (1986). Structural and functional characteristics of commissural neurons in the superior colliculus of the hamster. 253 197–215. 10.1002/cne.902530207 3793990

[B67] Roussel C. Erneux T. Schiffmann S. N. Gall D. (2006). Modulation of neuronal excitability by intracellular calcium buffering: From spiking to bursting. 39 455–466. 10.1016/j.ceca.2006.01.004 16530827

[B68] Schmidt M. Boller M. Özen G. Hall W. C. (2001). Disinhibition in rat superior colliculus mediated by GABAC receptors. 21 691–699. 10.1523/JNEUROSCI.21-02-00691.2001 11160448 PMC6763820

[B69] Shang C. Liu Z. Chen Z. Shi Y. Wang Q. Liu S. (2015). A parvalbumin-positive excitatory visual pathway to trigger fear responses in mice. 348 1472–1477. 10.1126/science.aaa8694 26113723

[B70] Soares J. G. Botelho E. P. Gattass R. (2001). Distribution of calbindin, parvalbumin and calretinin in the lateral geniculate nucleus and superior colliculus in *Cebus apella* monkeys. 22 139–146. 10.1016/S0891-0618(01)00123-5 11522436

[B71] Sohal V. S. Zhang F. Yizhar O. Deisseroth K. (2009). Parvalbumin neurons and gamma rhythms enhance cortical circuit performance. 459 698–702. 10.1038/nature07991 19396159 PMC3969859

[B72] Sooksawate T. Isa K. Behan M. Yanagawa Y. Isa T. (2011). Organization of GABAergic inhibition in the motor output layer of the superior colliculus. 33 421–432. 10.1111/j.1460-9568.2010.07535.x 21198984

[B73] Takahashi M. Sugiuchi Y. Izawa Y. Shinoda Y. (2005). Commissural excitation and inhibition by the superior colliculus in tectoreticular neurons projecting to omnipause neuron and inhibitory burst neuron regions. 94 1707–1726. 10.1152/jn.00347.2005 16105954

[B74] Takahashi M. Sugiuchi Y. Shinoda Y. (2010). Topographic organization of excitatory and inhibitory commissural connections in the superior colliculi and their functional roles in saccade generation. 104 3146–3167. 10.1152/jn.00554.2010 20926614

[B75] Taniguchi H. (2014). Genetic dissection of GABAergic neural circuits in mouse neocortex. 8:8. 10.3389/fncel.2014.00008 24478631 PMC3902216

[B76] Taniguchi H. He M. Wu P. Kim S. Paik R. Sugino K. (2011). A resource of cre driver lines for genetic targeting of GABAergic neurons in cerebral cortex. 71 995–1013. 10.1016/j.neuron.2011.07.026 21943598 PMC3779648

[B77] Tremblay R. Lee S. Rudy B. (2016). GABAergic interneurons in the neocortex: from cellular properties to circuits. 91 260–292. 10.1016/j.neuron.2016.06.033 27477017 PMC4980915

[B78] White L. E. Hodges H. D. Carnes K. M. Price J. L. Dubinsky J. M. (1994). Colocalization of excitatory and inhibitory neurotransmitter markers in striatal projection neurons in the rat. 339 328–340. 10.1002/cne.903390303 7907614

[B79] Whyland K. L. Masterson S. P. Slusarczyk A. S. Govindaiah G. Guido W. Bickford M. E. (2017). *Feedforward inhibitory Circuits within the Mouse Superior Colliculus. Program No. 228.08/CC21 2017 Neuroscience Meeting Planner*. Washington, DC: Society for Neuroscience.

[B80] Wöhr M. Orduz D. Gregory P. Moreno H. Khan U. Vörckel K. J. (2015). Lack of parvalbumin in mice leads to behavioral deficits relevant to all human autism core symptoms and related neural morphofunctional abnormalities. 5:e525. 10.1038/tp.2015.19 25756808 PMC4354349

[B81] Xu X. Callaway E. M. (2009). Laminar specificity of functional input to distinct types of inhibitory cortical neurons. 29 70–85. 10.1523/JNEUROSCI.4104-08.2009 PMC2656387 19129386

