# Supplementary figures and images for: Parvalbumin and GABA Microcircuits in the Mouse Superior Colliculus
Source: Front Neural Circuits. 2018 May 4;12:35. doi: 10.3389/fncir.2018.00035 (PMC5946669; doi:10.3389/fncir.2018.00035)

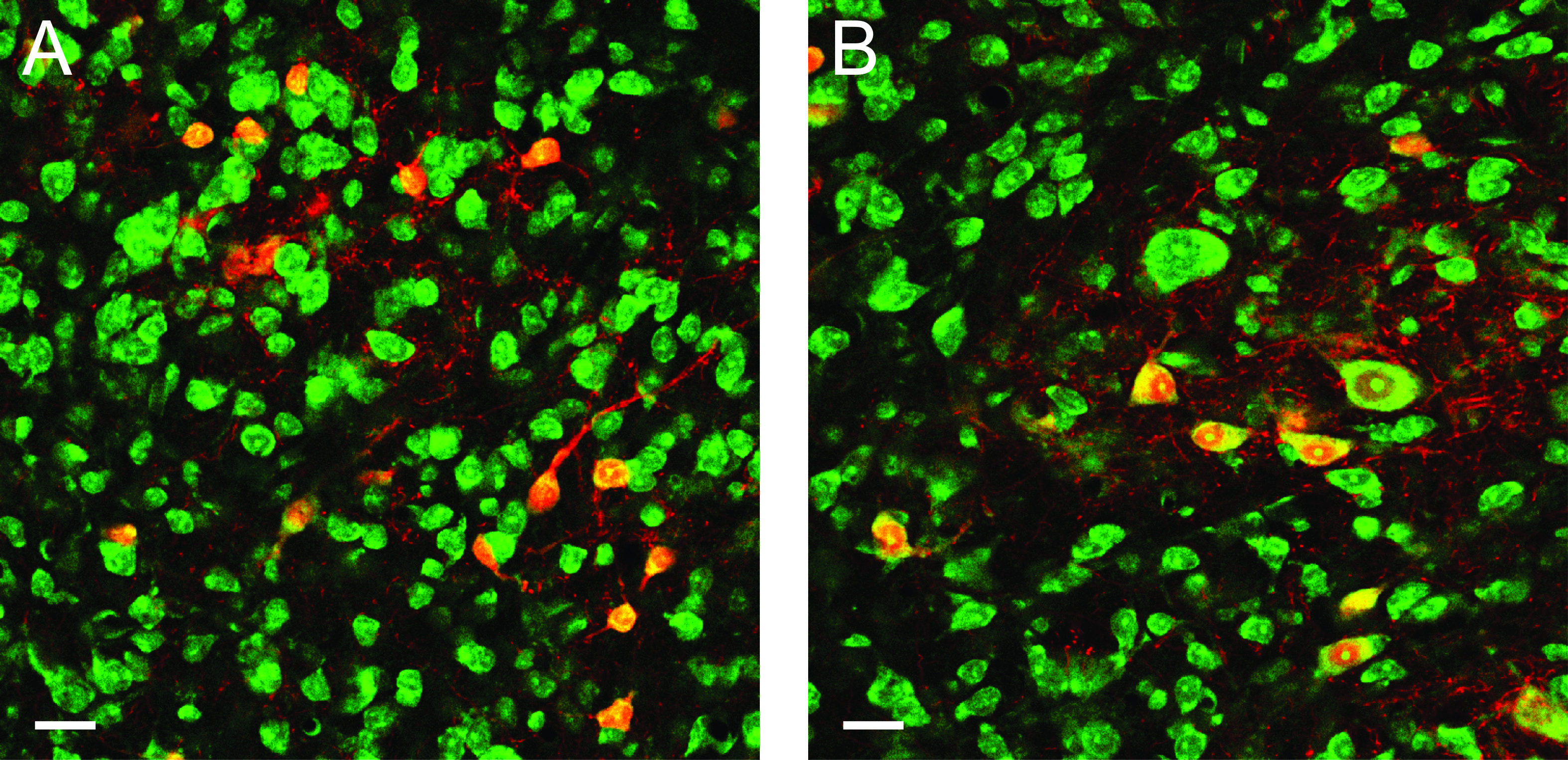

Supplement: FIGURE S1 — Histological sections through the sSC (A) and iSC (B) of an Ai9;PV-Cre mouse. Cells labeled with Neurotrace® appear in green (pseudo-colored) and PV+ cells (with tdTomato, red) appear as yellow. All PV+ cells co-localized with Neurotrace® indicating they are neurons. [file Image_1.JPEG]

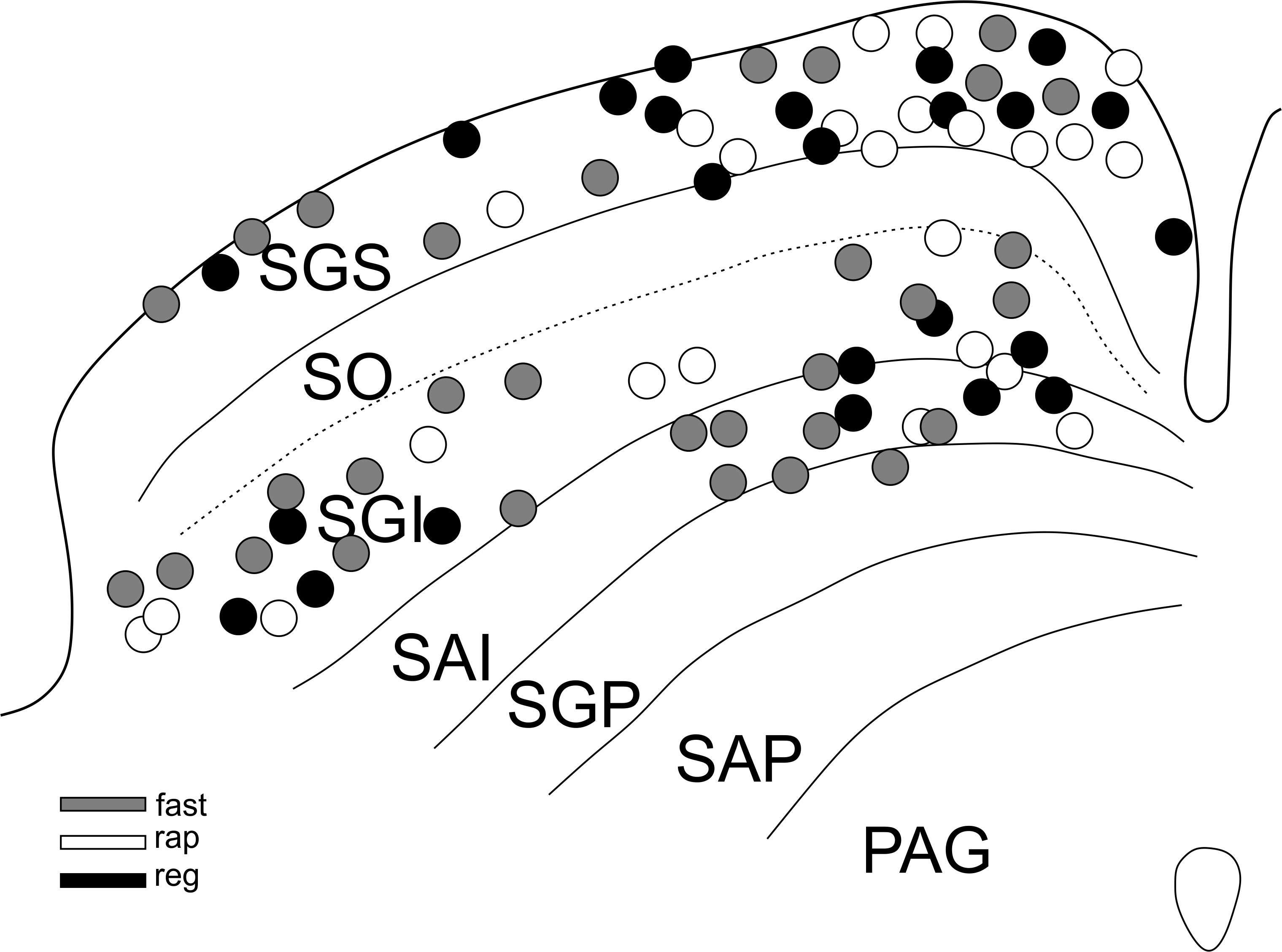

Supplement: FIGURE S2 — Distribution of PV+ neurons in the SC layers recorded in brain slices obtained from the Ai9;PV-Cre mouse. Each circle corresponds to individually recorded PV+ neurons and the color shows their respective electrophysiological phenotype (gray, fast spiking; white; rapidly inactivating; black, regular spiking). [file Image_2.JPEG]

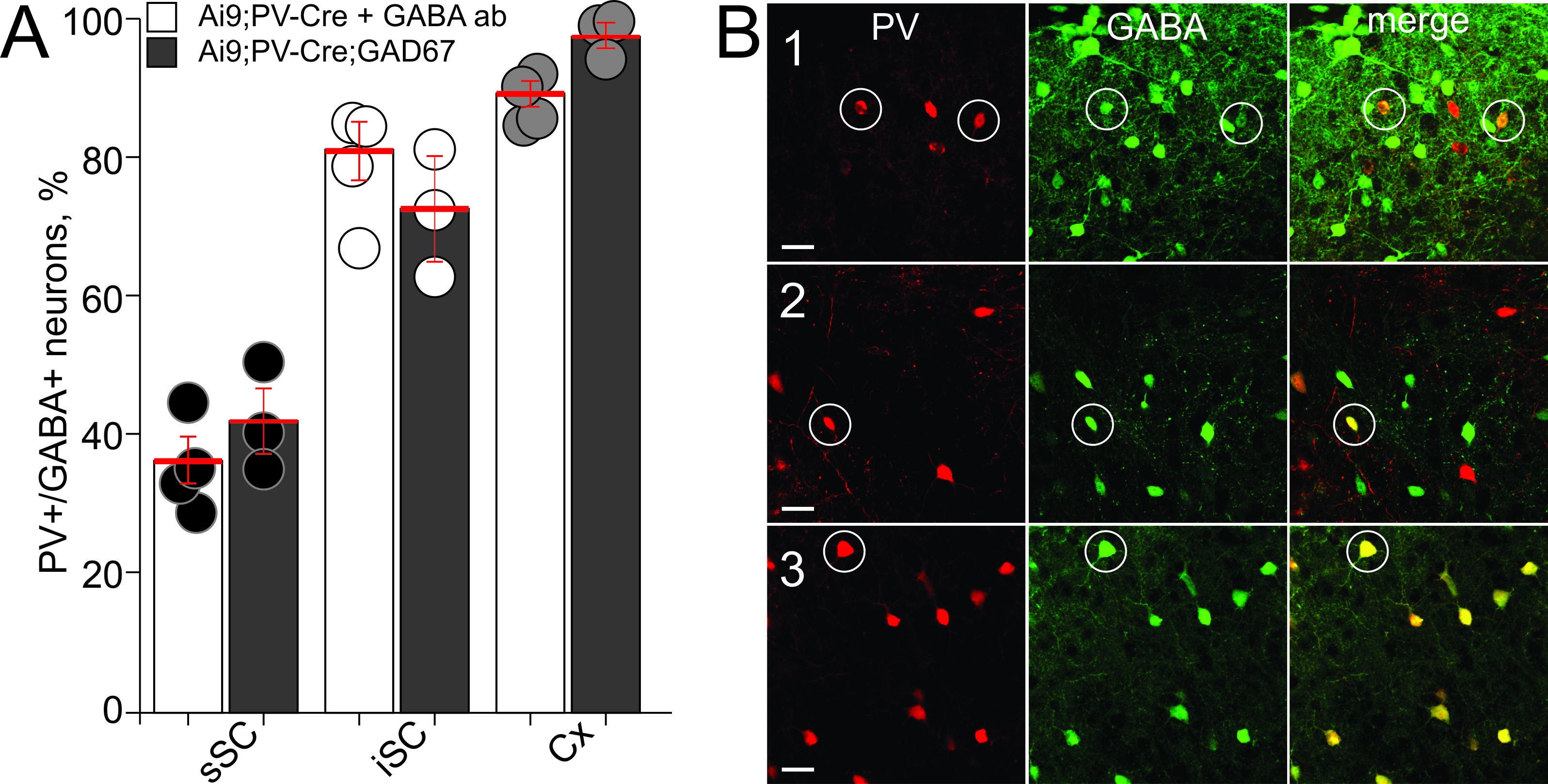

Supplement: FIGURE S3 — The percentage of PV+/GABA+ neurons from the total number of PV+ neurons in the Ai9;PV-Cre;GAD67 mouse are similar to those found in the Ai9;PV-Cre mouse using a GABA antibody. (A) Mean percentage of PV+/GABA+ vs. PV+ neurons calculated from slices obtained from the Ai9;PV-Cre mouse using a GABA antibody (white bars, Figure 2B) compared to the mean percentage of PV+/GABA+ neurons found in three Ai9;PV-Cre;GAD67 mice (gray bars); sSC = 42 ± 4% (black circles), iSC = 73 ± 7% (white circles), Cx = 98 ± 2%; n = 34 (gray circles). (B) PV+ (tdTomato), GABA+ (GFP) and merge panels showing high magnification confocal images from sections of sSC (B1), iSC (B2), and Cx (B3) obtained from an Ai9;PV-Cre;GAD67 mouse. Examples of double-labeled PV+/GABA+ neurons are highlighted on each of the images (white circles). Red bar: mean ± SE. [file Image_3.JPEG]

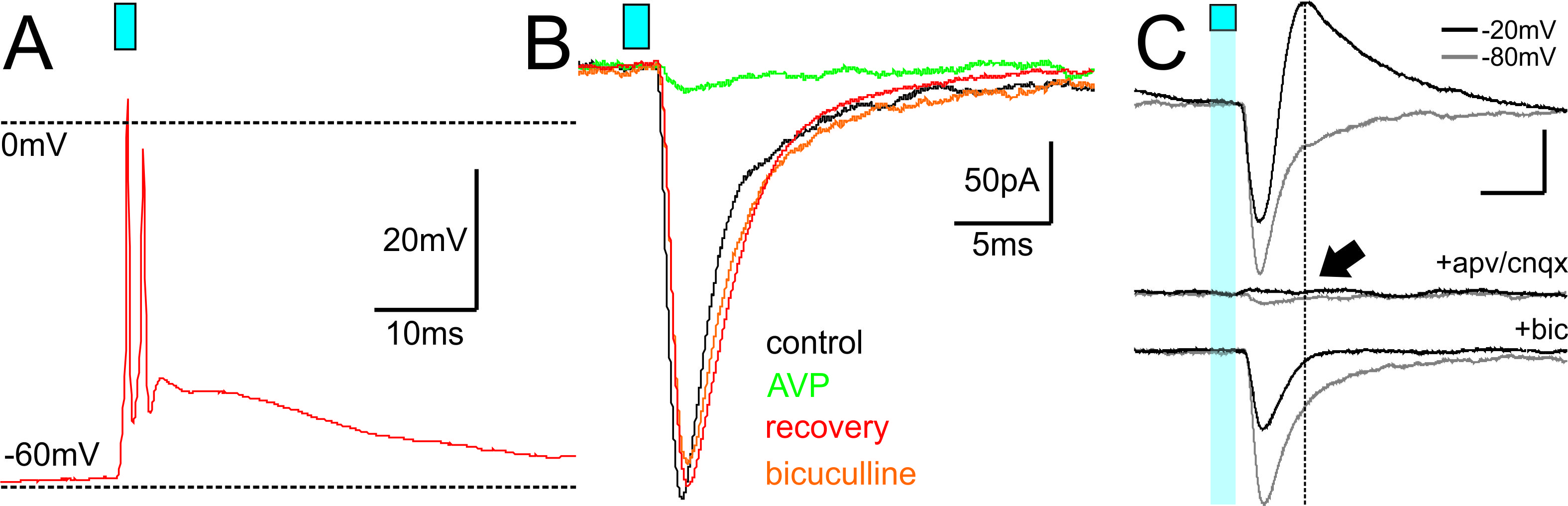

Supplement: FIGURE S4 — (A) Brief LED blue light pulses (cyan square, 2 ms) effectively evoked action potentials in PV+ neurons recorded in slices from Ai32;PV-Cre mice. Representative trace of a PV+ neuron (red trace) from sSC recorded in current-clamp configuration without QX-314 in the pipette (dashed lines, 0 mV and -60 mV). (B) Light-evoked EPSCs recorded at -80 mV (black trace) were blocked by APV/CNQX (50 μM/10 μM) (green trace). After ∼15 min washout, the EPSC amplitude was almost completely recovered (red trace). Bicuculline application (10 μM) did not affect the EPSC amplitude (orange trace). (C) Representative traces showing a PV+ driven inhibitory circuits classified as feedforward inhibition. Bath application of APV/CNQX diminished PSCs evoked at -80 and -20 mV. After washout, bicuculline selectively inhibited the outward current. Scale: 100 pA/5 ms. [file Image_4.JPEG]

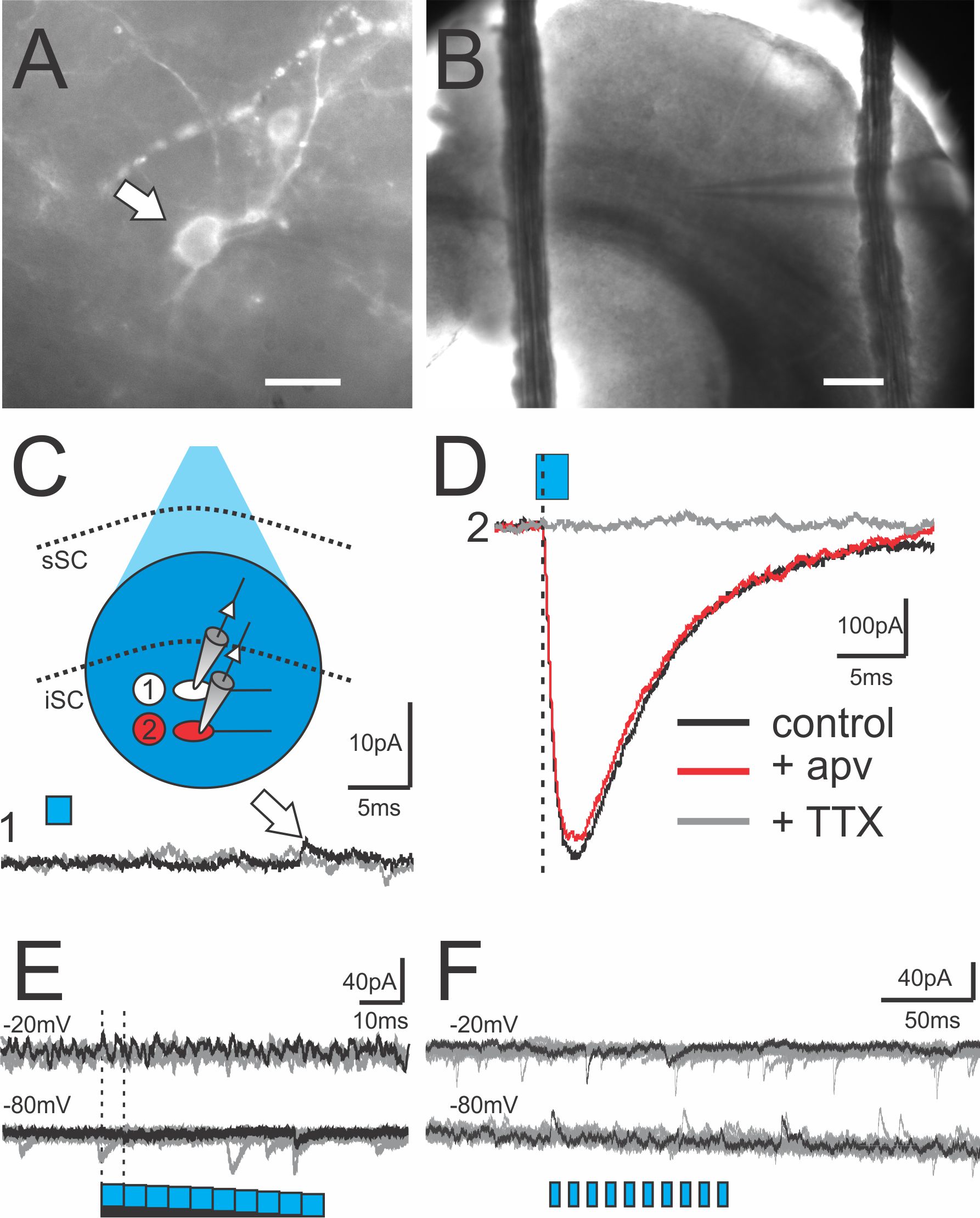

Supplement: FIGURE S5 — Optogenetic stimulation of PV+ neurons failed to evoke PSCs in PV- neurons recorded in iSC. (A) Fluorescent image (40×, GFP) showing a PV+ neuron in iSC (white arrow) of a brain slice from the Ai32;PV-Cre mouse. Scale: 20 μm. (B) DIC image showing the location of the recorded PV+ neuron. Scale: 200 μm. (C) Upper panel: Schematic illustration showing the recording of PV- (1) and PV+ (2) neurons in the iSC. Bottom panel: Representative traces of a recorded PV- neuron showing no significant current evoked upon 2 ms light stimulation in voltage-clamp configuration. Note the spontaneous IPSC evoked long after the light pulse when voltage is held at -20 mV (white arrow). Scale: 10 pA/5 ms. (D) Light stimulation elicited in a PV+ neuron a large and short latency APV/CNQX-insensitive inward current at -80 mV. Application of 1 μM TTX eliminated the inward current. The recording pipette contained QX-314 hence the absence of spikes. Scale: 100 pA/5 ms. (E) Light pulses of increasing length failed to evoke PSCs in iSC PV- neurons at either -80 mV or -20 mV Vm. (F) a train of 10 light pulses (5 ms in length) failed to evoke PSCs in iSC PV- neurons. Five consecutive traces are superimposed. [file Image_5.JPEG]
